# The influence of immunohistochemistry-based subtypes on overall survival in breast cancer spine metastases: a systematic review and meta-analysis

**DOI:** 10.1186/s12916-026-04715-0

**Published:** 2026-02-21

**Authors:** Fon-Yih Tsuang, Yun-Heng Li, Ting-Li Shen, Chiun-Sheng Huang, Chung Liang Chai

**Affiliations:** 1 Division of Neurosurgery, Department of Surgery, National Taiwan University Hospital, 1 Changde St, Taipei City, 100229 Taiwan; 2 Spine Tumor Center, National Taiwan University Hospital, 1 Changde St, Taipei City, 100229 Taiwan; 3 Department of Surgery, National Taiwan University Hospital, 1 Changde St, Taipei City, 100229 Taiwan; 4 Department of Neurosurgery, Yee Zen General Hospital, 30 Yangshin North Rd Ln 321, Yangmei, 32645 Taiwan; 5 School of Health Sciences, Faculty of Biology Medicine and Health, University of Manchester, Oxford Rd, Manchester, M13 9PL UK

**Keywords:** Breast cancer, Survival analysis, Metastasis, Spine, Subtype

## Abstract

**Background:**

Breast cancer spinal metastases present a growing clinical challenge, with survival outcomes varying significantly by immunohistochemistry-based subtype. Current prognostic models often overlook subtype-specific differences, potentially leading to suboptimal treatment decisions. This study aimed to establish the first comprehensive subtype-specific survival benchmarks for spinal metastases and to evaluate temporal trends in survival.

**Methods:**

We conducted a systematic review and meta-analysis of survival outcomes following a predefined protocol (PROSPERO CRD42024580279). Eligible studies reported overall survival (OS) in patients with breast cancer spinal metastases, stratified by immunohistochemistry-based subtype: hormone receptor (HR +), human epidermal growth factor receptor 2-enriched (HER2 +), and triple-negative breast cancer (TNBC). Survival data were extracted from published figures with a digitizer program and then processed in R. The median OS was analyzed through pooled survival curves with 95% confidence intervals, compared via log-rank tests. To evaluate temporal trends, we performed era-stratified analyses (pre-2000, 2000–2019, post-2020) using chronological partitioning of study enrollment periods.

**Results:**

After screening 2,348 records, we identified seven eligible cohorts comprising 672 patients. Analysis revealed significant survival differences among subtypes (log-rank *p* < 0.0001), with median OS of 28.9 months (95% CI 26.0–35.6; *n* = 347) for HR + , 43.7 months (31.9–48.7; *n* = 244) for HER2 + , and 10.7 months (8.9–19.2; *n* = 81) for TNBC (very low certainty of evidence for all outcomes). Temporal analysis of 4464 patients from 61 studies demonstrated significant survival improvements post-2020 (log-rank *p* < 0.0001).

**Conclusions:**

This study establishes the first real-world unadjusted survival reference for subtypes in breast cancer spinal metastases, suggesting a potential survival advantage of HER2 + /HR + over HER2 + /HR − tumors. These findings underscore the essential role of subtyping in refining prognostic assessment for spinal metastases. Our findings demonstrate a temporal improvement in survival and highlight the persistent need for contemporary data in this rapidly evolving clinical landscape.

**Trial registration:**

PROSPERO CRD42024580279.

**Supplementary Information:**

The online version contains supplementary material available at 10.1186/s12916-026-04715-0.

## Background

Breast cancer demonstrates significant genetic and clinical heterogeneity, with classification into immunohistochemistry-based subtypes based on hormone receptor (HR) status and human epidermal growth factor receptor 2 (HER2) expression. This stratification identifies three principal subtypes: HR +, HER2 +, and triple-negative breast cancer (TNBC) [1]. These profiles serve the dual critical purposes of prognostic stratification and therapeutic guidance. Of these subtypes, HR + demonstrates the highest prevalence and is consistently associated with the most favorable prognosis [1–3]. In contrast, HER2 + tumors occupy an intermediate prognostic position, while TNBC consistently demonstrates the poorest survival rates among all subtypes [1–3].

In metastatic breast cancer, immunohistochemistry-based profiling assumes prognostic importance. Kennecke et al. documented substantial median survival disparities among subtypes: HR + (26.4 months), HER2 +/HR + (19.2 months), HER2 +/HR − (8.4 months), and TNBC (6.0 months), highlighting the critical importance of immunohistochemistry-based classification in advanced breast cancer management [4].

When considering spinal metastases specifically, the literature grows notably sparse despite breast cancer representing the most common primary tumor among surgically treated spinal metastases, accounting for 18.5% of cases (358 of 1938) in a global cohort [5]. This paucity of data creates a significant knowledge gap, as surgical decision-making for spinal metastases depends heavily on accurate survival estimation. Patients with limited life expectancy may appropriately receive palliative interventions, while those with longer anticipated survival may be candidates for more definitive surgical interventions, such as en bloc resection or complex reconstruction.

Emerging evidence suggests immunohistochemistry-based profiling may surpass traditional prognostic systems like Tokuhashi in survival prediction [6]. However, current literature remains limited to small retrospective series or systematic reviews lacking pooled survival analyses [7]. For instance, Yao et al. identified only borderline significant survival differences between hormone-sensitive and non-sensitive subtypes (*p* = 0.056) in their Kaplan–Meier analysis [8].

While systemic therapeutic advances have substantially improved breast cancer outcomes overall [9, 10], their impact specifically on spinal metastases remains unclear. These improvements have paradoxically increased the prevalence of metastatic disease, including spinal involvement [10]. This systematic review addresses this critical knowledge gap by providing comprehensive, pooled survival data for each subtype, offering clinicians an evidence-based foundation for therapeutic decision-making in this complex patient population.

## Methods

This systematic review was reported in accordance with the PRISMA (Preferred Reporting Items for Systematic Reviews and Meta-Analysis) and AMSTAR (Assessing the methodological quality of systematic reviews) [11, 12]. All data are provided within the article and are freely available from the Open Science Framework [13]. The protocol was prospectively registered in the International Prospective Register of Systematic Reviews (PROSPERO; CRD42024580279) on August 27, 2024 [14]. In accordance with the TITAN 2025 [15] recommendations, artificial intelligence (ChatGPT, OpenAI GPT-4o) was used exclusively to assist with grammar refinement and readability. All writings were critically reviewed by the authors, who retain full responsibility for the content and accuracy of the manuscript.

Our study population was strictly defined as patients with histologically confirmed breast cancer metastatic to the spine. Subtyping was determined based on immunohistochemical analysis of the primary breast tumor, categorized according to established clinical criteria: HR + (estrogen receptor [ER]-positive and/or progesterone receptor [PR]-positive, HER2-negative), HER2 + (HER2-positive regardless of ER/PR expression), and triple-negative breast cancer (lacking expression of ER, PR, and HER2). In addition to these patient-level molecular subtypes, key study-level characteristics—such as the patient recruitment period—were also extracted and analyzed as covariates to account for potential temporal trends in management and outcomes. We excluded studies with unavailable survival data, those encompassing non-spinal metastases, and those failing to report total event numbers. Our analysis focused on clinical settings including tertiary hospitals and comprehensive cancer centers. While we accepted both randomized and non-randomized studies, inclusion required accessible survival data.

### Finding and assessing individual studies

**Fig. 1:**
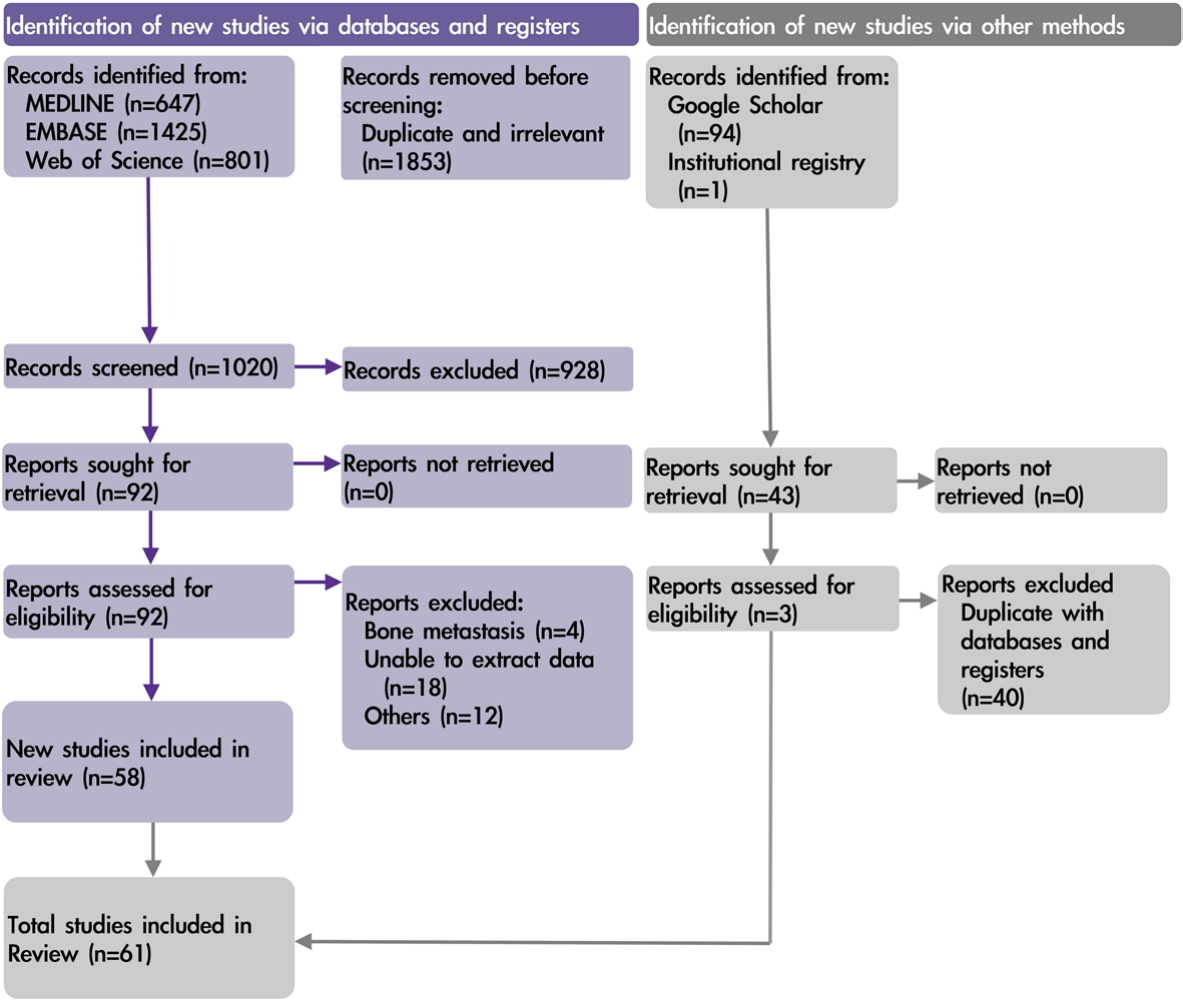
PRISMA 2020 flow chart

We searched Medline, Embase, Web of Science, and Google Scholar as illustrated in Fig. 1. We also incorporated a cohort of 142 patients from our institution (the Spine Oncology Registry of National Taiwan University Hospital). The search strategy for each database is detailed in Additional file 1. The search was performed on August 15, 2024. There were no restrictions on language. Study titles, abstracts, and full text were independently screened for inclusion by two authors (CLC and YHL), and discrepancies were resolved based on a consensus with a third author (FYT). To facilitate transparency and reproducibility, we used a pre-piloted form (Additional file 2) in the process of study inclusion and data extraction.

For qualitative assessment, the bias in the body of evidence was assessed according to the Grading of Recommendations, Assessment, Development, and Evaluation (GRADE) version dedicated to prognostic factors [16]. For the GRADE domain of study limitations (risk of bias), we assessed the quality of prognostic factor studies using the Quality in Prognosis Studies (QUIPS) tool (Additional file 3) [16–18]. The GRADE level was independently judged by CLC and YHL, and discrepancies were resolved based on a consensus with FYT.

### Synthesizing the body of evidence

Data extraction was straightforward for studies that provided the survival data of each patient within the publication [19]. For studies that provided a Kaplan–Meier curve, we used WebPlotDigitizer to obtain the approximate coordinates in the image [19, 20]. The coordinates were processed using the *IPDfromKM* package in R statistical software (R Development Core Team 2020, www.r-project.org) to obtain the survival data of each patient [21]. Survival time was measured in months.

The survival data of individuals were pooled to estimate the survival function separately for the HR +, HER2 +, and TNBC groups. To account for between-study heterogeneity, we employed a semi-parametric Cox proportional hazards mixed-effects model. Each study was included as a random intercept, allowing for study-specific baseline hazards while estimating pooled survival estimates. All included studies were systematically organized by their patient recruitment end dates to establish a chronological order. Studies with available subtype data were identified, enabling era-stratified survival analyses to investigate temporal trends in survival. Conventional definitions were used for confidence interval (95% CI) and *p-*value < 0.05 indicates significance for heterogeneity in frailty parameter θ and log-rank test. The Holm-Bonferroni-adjusted *p*-values are reported alongside raw *p*-values. The online GRADEpro GDT software was utilized for GRADE assessment. The R packages *coxme*, *frailtypack*, *survival*, *survRM2*, and *survminer* were used for analysis [22–25].

### Role of the funding source

The funder of the study had no role in the study design, data collection, data analysis, data interpretation, or writing of the report.

## Results

Of 2348 studies identified and screened (Fig. 1), of which 473 studies were assessed for eligibility and 61 were included in the meta-analysis. We documented the reasons for excluding 34 eligible studies (Additional file 4). Two studies were exclusively obtained from Google Scholar, one from the National Taiwan University Hospital Spine Oncology Registry, and the remaining 58 from either Medline or Embase. The inter-reviewer agreement using Cohen’s kappa was 0.91 (96% agreement; Additional file 5). Among the included studies, three were in German, and one in Danish; the rest were in English. Additional file 6 assesses the concordance between median survival time obtained through digital reconstruction of Kaplan–Meier curves and the corresponding numerically reported values in the source publications.

### Survival between subtypes

**Fig. 2:**
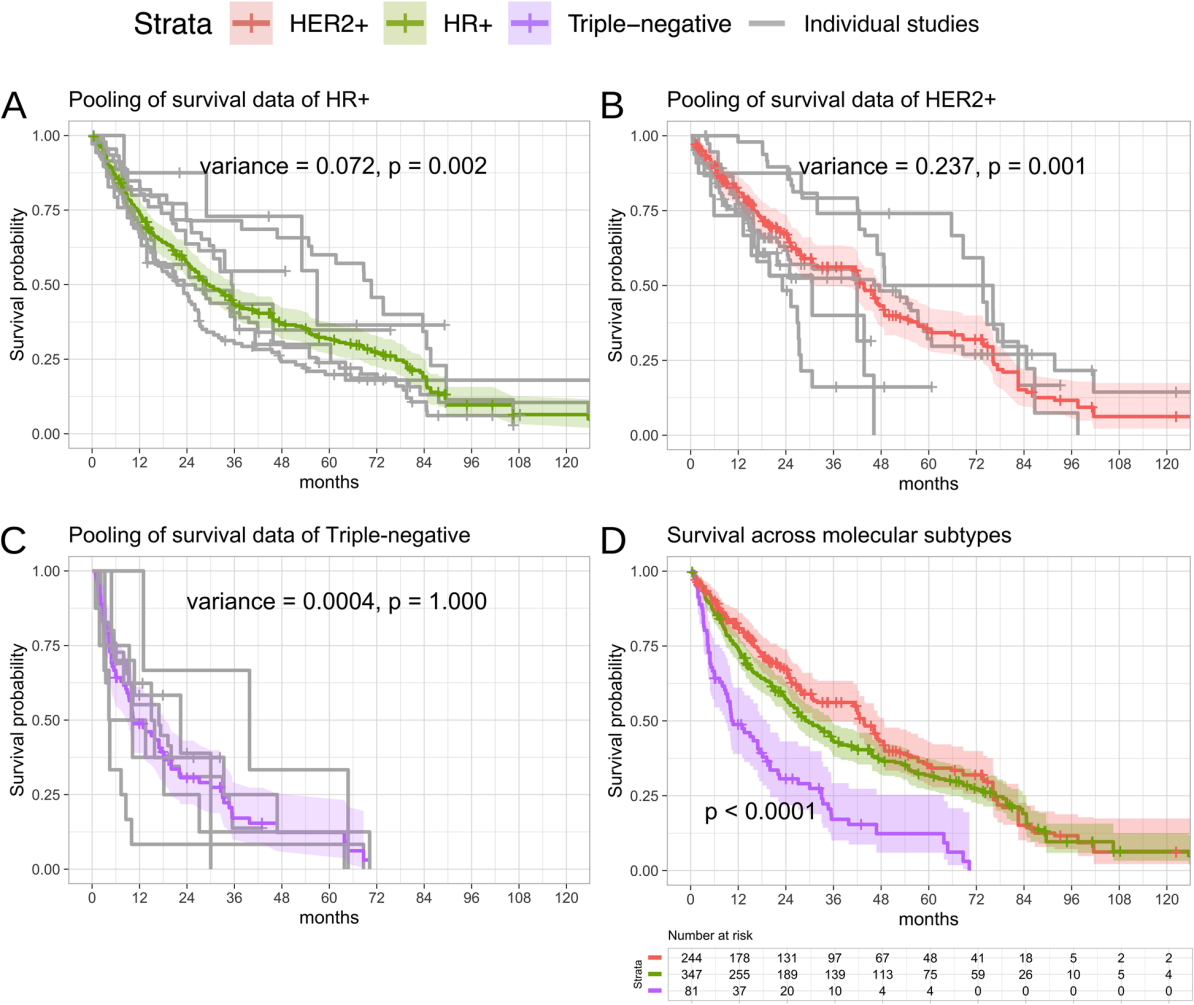
Pooled survival analysis by subtype in breast cancer spinal metastases. **A** HR + subtype survival curve. Solid line indicates Kaplan–Meier survival function with semi-transparent shading representing 95% confidence intervals. Frailty parameter analysis significant heterogeneity across studies (*p* = 0.002). **B** HER2-enriched (HER2 +) subtype survival curve. Frailty parameter analysis demonstrated significant heterogeneity across studies (*p* = 0.001). **C** Triple-negative breast cancer (TNBC) subtype survival curve. Frailty parameter analysis demonstrated non-significant heterogeneity across studies (*p* = 1.0). **D** Comparative survival analysis combining all subtypes (HR +, HER2 +, TNBC). Statistical comparison performed using log-rank test (*p*-value shown)

The characteristics of included studies, including individual citations, are detailed in Table 1. The Summary of Findings table and the pooled survival functions for HR +, HER2 +, and TNBC are presented in Table 2 and Fig. 2, respectively. For HR + (Fig. 2A), the pooled survival function, derived from multiple studies, estimated a median survival time of 28.9 months (95% CI, 26.0–35.6; 347 participants; 7 studies). Analysis of study-level heterogeneity using a Cox mixed-effects model revealed significant between-study variability (random effect variance = 0.072, *p* = 0.002). For HER2 + (Fig. 2B), the pooled analysis estimated a median survival time of 43.7 months (95% CI, 31.9–48.7; 244 participants; 6 studies). Substantial between-study heterogeneity was observed in the HER2 + group (random effect variance = 0.237, *p* = 0.001). For TNBC (Fig. 2C), the median survival time was 10.7 months (95% CI, 8.87–19.2; 81 participants; 7 studies). The TNBC subgroup demonstrated negligible between-study variability (random effect variance = 0.0004, *p* = 1.000).

**Table 1:** Characteristics of included studies with extractable profiling data

Study ID	Institute	Cohort span	Treatment	Mean age (SD)
Amelot 2020 [2]	Batiment Babinski Groupe Hospitalier Pitié-Salpêtrière, Paris	2014–2017	19/123 (15.4%) decompressive laminectomy + posterior fixation; 76/123 (61.8%) radiotherapy; 28/123 (22.7%) not reported	59.8 (11.0)
Duvall 2023 [26]	Massachusetts General Hospital, Boston	2008–2021	83/98 (84.7%) separation surgery; 26/98 (27%) corpectomy	56.4 (12.1)
NTUH	National Taiwan University Hospital, Taipei	2008–2024	63/142 (44.4%) debulking; 71/142 (50%) palliation surgery; 5/142 (3.5%) vertebrec-/spondylec-tomy; 3/142 (2.1%) vertebroplasty	55.0 (10.4)
Soto 2023 [27]	Mexico National Cancer Institute, Mexico City	2011–2017	42/56 (75%) received radiotherapy; 14/56 (25%) not reported	53.6 (12.6)
Wang 2014 [28]	Aarhus University Hospital and Copenhagen University Hospital Rigshospitalet, Denmark	1992–2011	18/151 (11.9%) decompression-only; 64/151 (42.4%) decompression + posterior fixation; 29/151 (19.2%) decompression + posterior fixation + reconstruction; 18/151 (11.9%) vertebrectomy + 360° reconstruction; 22/151 (14.6%) irretrievable treatments	58.7 (11.3)
Yao 2022 [8]	Affiliated Cancer Hospital of Zheng Zhou University, He Nan Cancer Hospital, Zheng Zhou	2017–2020	32/54 (59.3%) palliative decompression; 21/54 (38.9%) vertebrae piecemeal curettage; 1/54 (1.9%) total vertebral en bloc resection	51.3 (8.6)
Zhao 2018 [29]	Changzheng Hospital, Second Military Medical University, Shanghai	2005–2015	8/87 (9.2%) percutaneous vertebroplasty; 75/87 (86.2%) vertebrectomies + reconstruction; 3/87 (3.4%) laminectomies + reconstruction	52 (10.3)

**Table 2:** Summary of findings

Outcomes	Anticipated absolute number of patients survival* (95% CI)			Median overall survival (95% CI)	No. of participants (studies)	Certainty of the evidence (GRADE)
	**1-year**	**5-year**	**10-year**			
**HR + ** Hormone receptor positive, HER2 negative	**7.4 per 10** (7.0 to 7.9)	**3.2 per 10** (2.7 to 3.8)	**0.6 per 10** (0.3 to 1.3)	**28.9 months** (26.0 to 35.6)	347 (7 studies)	⨀◯◯◯ Very low
**HER2 + ** Human epidermal growth factor receptor 2 positive	**8.1 per 10** (7.6 to 8.6)	**3.5 per 10** (2.9 to 4.3)	**0.6 per 10** (0.2 to 1.7)	**43.7 months** (31.9 to 48.7)	244 (6 studies)	⨀◯◯◯ Very low
**Triple negative** Negative hormone receptor and HER2	**4.9 per 10** (3.9 to 6.1)	**1.2 per 10** (0.6 to 2.5)	**0 per 10** (- to -)	**10.7 months** (8.87 to 19.2)	81 (7 studies)	⨀◯◯◯ Very low

GRADE: grading of recommendations, assessment, development, and evaluation

*CI* confidence interval

*The absolute data is estimated from the percentage of survival (and its 95% confidence interval)

#### GRADE Working Group grades of evidence risk associated with the prognostic factor

- High certainty: We are very confident that the variation in risk associated with the prognostic factor (probability of future events in those with/without the prognostic factor) lies close to that of the estimate.
- Moderate certainty: We are moderately confident that the variation in risk associated with the prognostic factor (probability of future events in those with/without the prognostic factor) is likely to be close to the estimate, but there is a possibility that it is substantially different.
- Low certainty: Our certainty in the estimate is limited: the variation in risk associated with the prognostic factor (probability of future events in those with/without the prognostic factor) may be substantially different from the estimate.
- Very low certainty: We have very little certainty in the estimate: the variation in risk associated with the prognostic factor (probability of future events in those with/without the prognostic factor) is likely to be substantially different from the estimate.

**Fig. 3:**
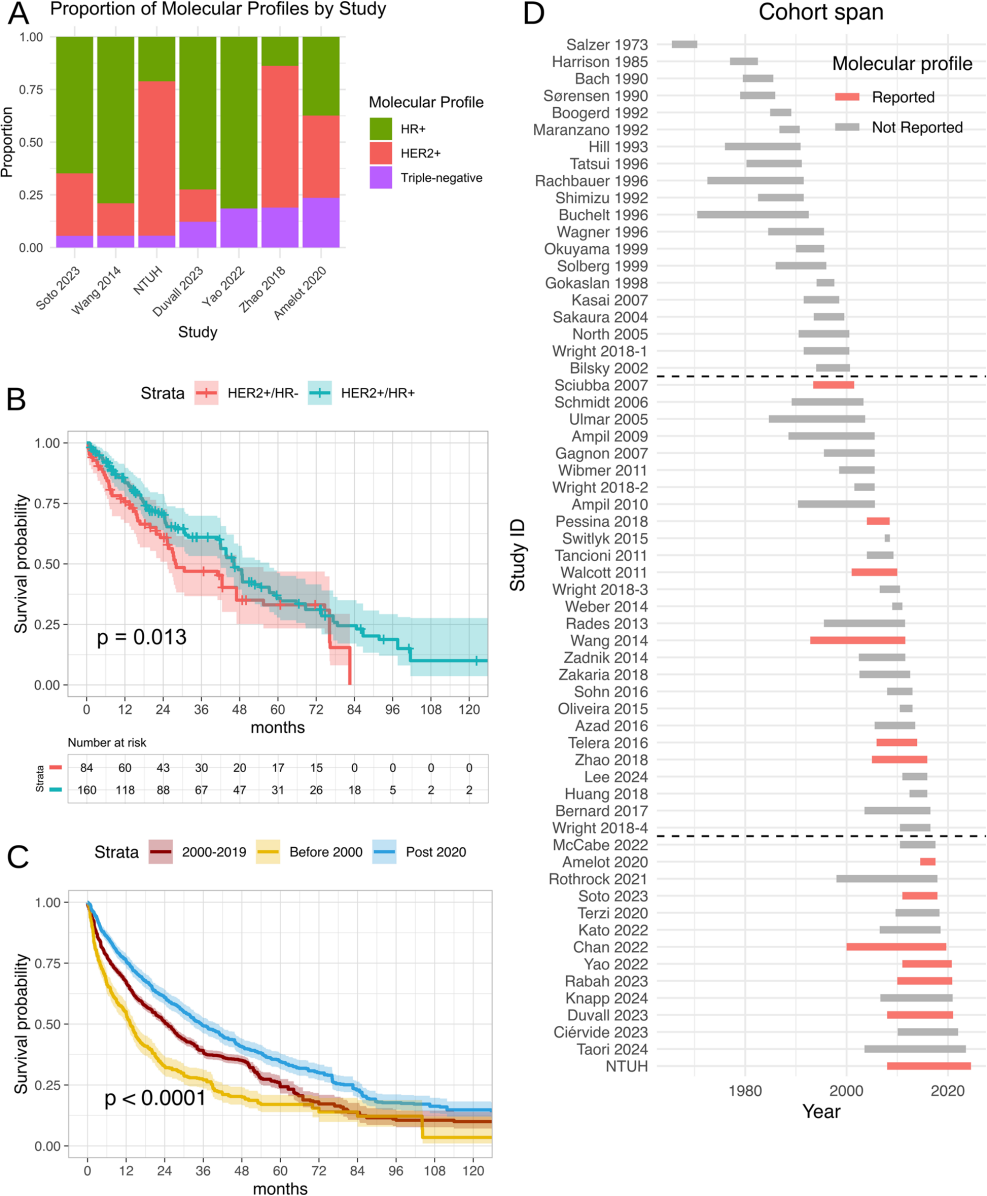
Meta-analysis of subtypes and temporal survival trends in breast cancer spinal metastases. **A** Distribution of subtypes across included studies. Vertical bar chart displays proportional variations in subtype representation (HR +, HER2 +, TNBC) among included studies. **B** Comparative Kaplan–Meier survival analysis between HER2 +/HR + and HER2 +/HR − subtypes. Statistical significance assessed via log-rank test (*p*-value shown). **C** Temporal survival improvement analysis. Survival outcomes were stratified using a two-tiered partitioning system (see panel **D**). Statistical comparison performed with log-rank test (*p*-value shown). **D** Study enrollment timeline. Horizontal bars depict study periods (*x*-axis: calendar years), with bar lengths corresponding to study duration. Partitions are indicated by dashed horizontal lines: pre-2000 (upper third), 2000–2019 (middle third), and post-2020 (lower third). Studies reporting subtype data are highlighted in red (see legend). Abbreviation: NTUH, National Taiwan University Hospital

Our Kaplan–Meier analysis of 672 patients from seven studies (Fig. 2D) revealed survival differences among subtypes (log-rank raw *p* < 0.0001; Holm-adjusted *p* < 0.0001). As shown in Fig. 3A, the proportional representation of subtypes varied across contributing studies. This methodological context is crucial for interpreting the observed survival patterns, particularly when comparing results to other heterogeneous cohorts.

To further characterize survival disparities within the HER2 + population, we compared outcomes between the HER2 +/HR − and HER2 +/HR + cohorts. After adjusting for study-level heterogeneity using a shared frailty Cox model, patients with HER2 +/HR + exhibited longer survival compared to the HER2 +/HR − group (HR = 0.54; 95% CI: 0.36–0.81; raw *p* = 0.003; Holm-adjusted *p* = 0.006), representing a 46% reduction in the risk of mortality. Consistent with these findings, the meta-analysis of individual hazard ratios yielded HR = 0.53; 95% CI: 0.35–0.80; *I*^2^ = 44.1%; raw *p* = 0.003; Holm-adjusted *p* = 0.006; 5 studies (see Additional file 7 for the forest plot). Furthermore, the Kaplan–Meier estimates demonstrated distinct survival separation (*p* = 0.013, Fig. 3B). Restricted mean survival time analysis over a 120-month horizon revealed a clinically meaningful benefit for the HER2 +/HR + cohort, with an absolute gain of 11.8 months in mean survival (95% CI: 2.08–21.6; raw *p* = 0.017; Holm-adjusted *p* = 0.034). Although post hoc power was 77.5% (slightly below the conventional 80%), the consistent findings across multiple analytical methods support the robustness of the results. We acknowledge that sample size remains a constraint, and these results warrant validation in larger, prospective cohorts.

### Survival changes over time

Our Kaplan–Meier analysis of 4464 patients from 61 studies revealed a statistically significant temporal improvement beginning with studies published in 2020 (Fig. 3C; log-rank raw *p* < 0.0001, Holm-adjusted *p* < 0.0001). This observation was corroborated by meta-regression, in which later median year of enrollment was independently associated with improved outcomes (hazard ratio per calendar year 0.98, 95% CI 0.96–0.98; raw *p* < 0.0001, Holm-adjusted *p* < 0.0001), corresponding to an estimated 2.4% reduction in hazard of death per year. The characteristics of included studies are detailed in Additional file 8 [5, 30–79]. We implemented a two-tiered partitioning system (Fig. 3D), with an initial stratification based on the study by Sciubba et al. (2007) [49], which was the first to incorporate subtyping and secondary stratification at 2020, marking a period in which recent therapeutic advances [80, 81] were more consistently reflected in survival data and subtyping was more widely reported. This post hoc classification scheme, while requiring prospective validation, has been fully documented with all supporting data deposited in the Open Science Framework to enable community evaluation and iterative improvement [13].

**Fig. 4:**
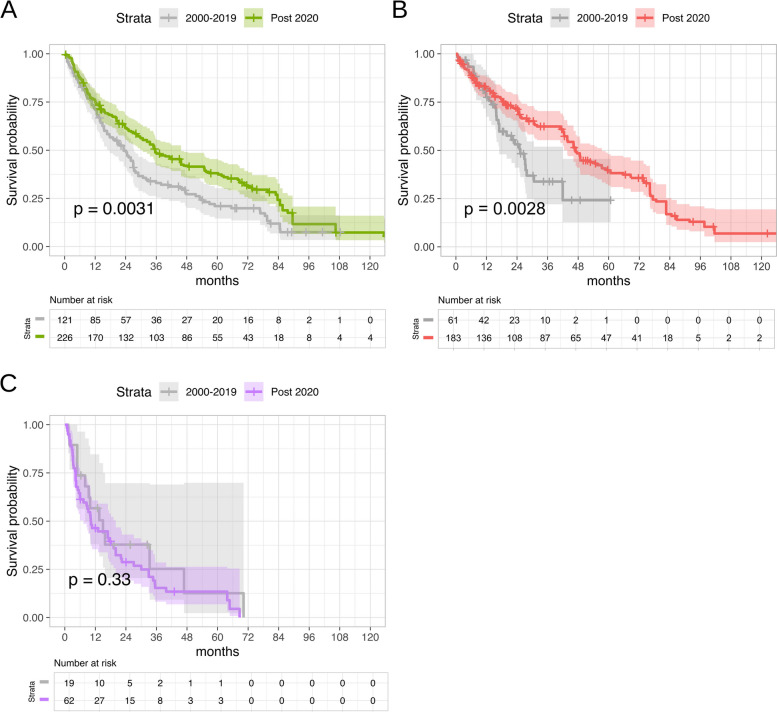
Comparative survival analysis by subtype across treatment eras. Kaplan–Meier curves demonstrate temporal survival improvements between 2000–2019 and post-2020 for **A** HR +, **B** HER2 +, and **C** TNBC. Statistical comparisons were performed using log-rank tests (*p*-values shown for each subtype)

Building upon this temporal partitioning framework, we conducted subtype-specific survival analyses. Significant survival improvements in the post-2020 cohort were observed for both HR + (Fig. 4A; log-rank test, raw *p* = 0.0031, Holm-adjusted *p* = 0.0062; statistical power = 0.85) and HER2 + (Fig. 4C; log-rank test, raw *p* = 0.0028, Holm-adjusted *p* = 0.0056; statistical power = 0.97) subtypes, indicating that these findings are well-supported by sufficient statistical power. For TNBC, no statistically significant temporal difference was observed (Fig. 4C; log-rank test, raw *p* = 0.33, Holm-adjusted *p* = 0.66; statistical power = 0.16), a finding that may reflect limited statistical power in this smaller subgroup.

Based on the restricted mean survival time analysis, significant improvements in survival were observed for patients treated in the post-2020 era compared to the 2000–2019 period, though the magnitude and significance varied by subtype. In the HR + subgroup, the post-2020 cohort survived an average of 11.9 months longer within the first 108 months (95% CI: 4.14–19.67; *P* = 0.003). Similarly, HER2 + patients experienced a 10.2-month increase in average survival over the first 60 months (95% CI: 3.46–16.90; *P* = 0.003). Conversely, no significant temporal survival difference was detected in the TNBC subgroup, with a non-significant restricted mean survival time difference of − 3.7 months (95% CI: − 14.90–7.45; *P* = 0.514) over 60 months.

### GRADE assessment

The GRADE scores of all outcomes indicated very low certainty of evidence (Table 2 and Additional file 3). The overrepresentation of surgically managed patients from high-resource tertiary referral centers introduces three critical selection biases: (1) inherent selection of optimal surgical candidates, (2) socioeconomic stratification affecting access to advanced diagnostics and therapies, and (3) institutional variability in palliative care protocols. These factors collectively constrain the external validity of our survival estimates, particularly for non-surgical patients and non-specialized centers.

## Discussion

In this systematic review and meta-analysis, we present the first unadjusted real-world Kaplan–Meier survival curves for patients with breast cancer spinal metastases stratified by subtype. Our findings are consistent with and extend the data from large national cohorts. For example, while our pooled median survival for all HER2 + was 43.7 months, the National Cancer Database (2010–2013) focusing on HER2 subgroups show a significant disparity between HER2 +/HR + and HER2 +/HR − from any site, with median survival of 56.7 and 21.5 months, respectively [82]. Our spine-specific study corroborates this prognostic stratification, demonstrating a statistically significant difference in patients with spinal metastases: median survival of 46.2 months (95% CI: 42.0–57.3) for HER2 +/HR + versus 28.1 (95% CI: 25.3–47.0) for HER2 +/HR − disease. The numerically lower survival in both subgroups compared to the all-site database likely reflects the specific prognostic impact of symptomatic spinal involvement. This divergence in outcomes aligns with the distinct metastatic patterns described for these subtypes, where HER2 +/HR + disease demonstrates a predilection for bone, while HER2 +/HR − disease more frequently involves visceral sites such as brain, liver, and lung [82]. Consequently, HER2 +/HR + patients are disproportionately represented in spine metastasis cohorts. This has direct clinical implications: for the spine specialist, recognizing this substantial survival difference within HER2-positive patients is crucial, as the markedly longer anticipated survival in the HER2 +/HR + subgroup must inform the goals and aggressiveness of local spinal intervention.

Although a temporal improvement in survival might be expected, our analysis specifically addresses the lack of robust evidence for this trend in the context of breast cancer spinal metastases. For instance, prior large-scale analyses by Rothrock et al. (*n* = 149) and Wright et al. (*n* = 359) specifically attempted but failed to demonstrate a clear temporal survival benefit in similar spine metastasis populations [5, 72]. Therefore, our finding of a significant era-stratified survival improvement (pre-2000 vs. 2000–2019 vs. post-2020) provides novel, quantitative evidence for this evolving clinical paradigm.

Our findings also underscore the importance of using contemporary data for survival estimation. Breast cancer outcomes have improved substantially in recent years due to therapeutic advances [9]. It is therefore crucial to use contemporary data in prognostic modeling. The National Cancer Database [82], collected prior to 2013, were considered outdated for clinical decision-making. Applying historical survival estimates to contemporary patients risks underestimating life expectancy, potentially favoring overly conservative interventions. Our findings provide a timely reference for subtype-specific survival, emphasizing the persistent requirement for up-to-date survival data.

### Future implications

This study highlights a critical reporting gap: subtype-stratified survival outcomes were available for only 15% of patients across the included literature. Such limited reporting suggests a persistent underappreciation of subtyping as a prognostic tool in spinal metastases management. Additionally, time-to-metastasis patterns may vary significantly across subtypes, warranting further investigation. We anticipate that these findings will encourage more rigorous documentation and subtype-specific survival analyses in future research.

### Limitations

Several methodological limitations should be acknowledged. First, survival times were reconstructed from published Kaplan–Meier curves rather than original patient-level data, introducing potential discrepancies. Second, while multinational pooling provided real-world insights and greater statistical power, it lacked individual-level covariates and assumed patient independence, overlooking clustering effects. Institutional variation in patient selection, treatment protocols, and era effects remains embedded in these results. Together, these factors explain the very low certainty GRADE rating assigned to all outcomes. Our analytic strategy prioritized breadth over precision—a deliberate trade-off reflected in our cautious interpretations. Moreover, frailty models require large cluster sizes to detect heterogeneity with adequate power; in our dataset, each subtype was represented by only 5–7 study arms, limiting power to assess between-study variability. Finally, most included cohorts disproportionately represent surgically treated patients from high-resource tertiary centers, restricting the generalizability of our findings.

## Conclusions

This study establishes the first unadjusted real-world survival reference for subtypes in breast cancer spinal metastases, demonstrating a potential survival advantage of HER2 +/HR + over HER2 +/HR − tumors. Our results underscore the critical importance of immunohistochemical subtyping in prognosis and treatment planning, demonstrate a significant temporal improvement in survival, and highlight the need for contemporary data in this rapidly evolving field. Future research must adopt standardized immunohistochemistry-based classification and reporting methods to enable meaningful comparisons and optimize treatment algorithms.

## Supplementary Information

Additional file 1: Search Strategy for studies included in this review.

Additional file 2: Data extraction form.

Additional file 3: GRADE definitions and judgment process.

Additional file 4: Reasons for excluding 34 eligible studies.

Additional file 5: Study selection and inter-assessor agreement.

Additional file 6: Median overall survival: digitized vs. reported estimates.

Additional file 7: Forest plot of the hazard ratio for HER2 +/HR + compared to HER2 +/HR-.

Additional file 8: Characteristics of included studies for survival changes over time.

## Data Availability

All data are provided within the article and are freely available from the Open Science Framework with publication. URL: https://osf.io/nfx7w

## Abbreviations

AMSTAR: Assessing the methodological quality of systematic reviews
CI: Confidence interval
CLC: Chung Liang Chai
FYT: Fon-Yih Tsuang
GRADE: Grading of Recommendations Assessment, Development and Evaluation
HER2 +: Human epidermal growth factor receptor 2-enriched
HR +: Hormone receptor positive
HR: Hazard ratio
NTUH: National Taiwan University Hospital
OS: Overall survival
PRISMA: Preferred Reporting Items for Systematic Reviews and Meta-Analysis
PROSPERO: International Prospective Register of Systematic Reviews
QUIPS: Quality in Prognosis Studies
RMST: Restricted mean survival time
SD: Standard deviation
TITAN: Transparency In The reporting of Artificial Intelligence
TNBC: Triple-negative breast cancer
YHL: Yun-Heng Li

## References

[1] Orrantia-Borunda E, Anchondo-Nuñez P, Acuña-Aguilar LE, Gómez-Valles FO, Ramírez-Valdespino CA. Subtypes of Breast Cancer. In: Department of Medical Education, Dr. Kiran C. Patel College of Allopathic Medicine, Nova Southeastern University, FL, USA, Mayrovitz HN, editors. Breast Cancer. Exon Publications; 2022. p. 31–42. 10.36255/exon-publications-breast-cancer-subtypes. 36122153

[2] Amelot A, Terrier L-M, Cristini J, Buffenoir K, Pascal-Moussellard H, Carpentier A, et al. Survival in breast cancer patients with spine metastases: prognostic assessment involving molecular markers. Eur J Surg Oncol J Eur Soc Surg Oncol Br Assoc Surg Oncol. 2020;46:1021–7. 10.1016/j.ejso.2019.12.026. 10.1016/j.ejso.2019.12.026 31899046

[3] Laurberg T, Alsner J, Tramm T, Jensen V, Lyngholm CD, Christiansen PM, et al. Impact of age, intrinsic subtype and local treatment on long-term local-regional recurrence and breast cancer mortality among low-risk breast cancer patients. Acta Oncol. 2017;56:59–67. 10.1080/0284186X.2016.1246803. 27846764 10.1080/0284186X.2016.1246803

[4] Kennecke H, Yerushalmi R, Woods R, Cheang MCU, Voduc D, Speers CH, et al. Metastatic Behavior of Breast Cancer Subtypes. J Clin Oncol. 2010;28:3271–7. 10.1200/JCO.2009.25.9820. 20498394 10.1200/JCO.2009.25.9820

[5] Wright E, Ricciardi F, Arts M, Buchowski J, Chung C, Coppes M, et al. Metastatic spine tumor epidemiology: comparison of trends in surgery across two decades and three continents. World Neurosurgery. 2018;114:E809. 10.1016/j.wneu.2018.03.091. 29572177 10.1016/j.wneu.2018.03.091

[6] Tan K-A, Tan JH, Zaw AS, Tan JYH, Hey HWD, Kumar N. Evaluation of prognostic factors and proposed changes to the modified Tokuhashi score in patients with spinal metastases from breast cancer. Spine. 2018;43:512–9. 10.1097/BRS.0000000000002350. 28749856 10.1097/BRS.0000000000002350

[7] Sciubba DM, Goodwin CR, Yurter A, Ju D, Gokaslan ZL, Fisher C, et al. A systematic review of clinical outcomes and prognostic factors for patients undergoing surgery for spinal metastases secondary to breast cancer. Glob Spine J. 2016;6:482–96. 10.1055/s-0035-1564807. 10.1055/s-0035-1564807 PMC4947406 27433433

[8] Yao W, Li Z, Guo L, Niu L, Yan M, Niu X. Surgical efficacy and prognosis of 54 cases of spinal metastases from breast cancer. World Neurosurg. 2022;165:e373. 10.1016/j.wneu.2022.06.060. 35750145 10.1016/j.wneu.2022.06.060

[9] Miglietta F, Bottosso M, Griguolo G, Dieci MV, Guarneri V. Major advancements in metastatic breast cancer treatment: when expanding options means prolonging survival. ESMO Open. 2022;7:100409. 10.1016/j.esmoop.2022.100409. 35227965 10.1016/j.esmoop.2022.100409 PMC8886005

[10] Qiao R, Zhang H-R, Ma R-X, Li R, Hu Y. Prognostic factors for bone survival and functional outcomes in patients with breast cancer spine metastases. Technol Cancer Res Treat. 2022;21:15330338221122642. 10.1177/15330338221122642. 36214255 10.1177/15330338221122642 PMC9551339

[11] Shea BJ, Reeves BC, Wells G, Thuku M, Hamel C, Moran J, et al. AMSTAR 2: a critical appraisal tool for systematic reviews that include randomised or non-randomised studies of healthcare interventions, or both. BMJ. 2017:j4008. 10.1136/bmj.j4008. 10.1136/bmj.j4008 PMC5833365 28935701

[12] Page MJ, McKenzie JE, Bossuyt PM, Boutron I, Hoffmann TC, Mulrow CD, et al. The PRISMA 2020 statement: an updated guideline for reporting systematic reviews. BMJ. 2021;372:n71. 10.1136/bmj.n71. 33782057 10.1136/bmj.n71 PMC8005924

[13] Chai CL. The Influence of molecular subtypes on overall survival in breast cancer spine metastasis: A systematic review and meta-analysis. Open Science Framework. https://osf.io/nfx7w. 10.1186/s12916-026-04715-0 PMC13032407 41723490

[14] Tsuang F-Y, Chai CL. The Influence of Molecular Subtypes on Overall Survival in Breast Cancer Spine Metastasis: A Systematic Review and Meta-analysis. PROSPERO International prospective register of reviews. https://www.crd.york.ac.uk/PROSPERO/view/CRD42024580279.

[15] Agha R, Mathew G, Rashid R, Kerwan A, Al-Jabir A, Sohrabi C, et al. Transparency In The reporting of Artificial INtelligence – the TITAN guideline. Prem J Sci. 2025. 10.70389/PJS.100082.

[16] Foroutan F, Guyatt G, Zuk V, Vandvik PO, Alba AC, Mustafa R, et al. GRADE guidelines 28: use of GRADE for the assessment of evidence about prognostic factors: rating certainty in identification of groups of patients with different absolute risks. J Clin Epidemiol. 2020;121:62–70. 10.1016/j.jclinepi.2019.12.023. 31982539 10.1016/j.jclinepi.2019.12.023

[17] Hayden JA, Van Der Windt DA, Cartwright JL, Côté P, Bombardier C. Assessing bias in studies of prognostic factors. Ann Intern Med. 2013;158:280–6. 10.7326/0003-4819-158-4-201302190-00009. 23420236 10.7326/0003-4819-158-4-201302190-00009

[18] Murad MH, Mustafa RA, Schünemann HJ, Sultan S, Santesso N. Rating the certainty in evidence in the absence of a single estimate of effect. Evid Based Med. 2017;22:85–7. 10.1136/ebmed-2017-110668. 28320705 10.1136/ebmed-2017-110668 PMC5502230

[19] Tsuang F-Y, Jeon JP, Huang A-P, Chai CL. Overall survival of non-small cell lung cancer with spinal metastasis: a systematic review and meta-analysis. Neurospine. 2023;20:567–76. 10.14245/ns.2245026.513. 37401075 10.14245/ns.2245026.513 PMC10323357

[20] Rohatgi A. WebPlotDigitizer. 2021.

[21] Liu N, Zhou Y, Lee JJ. Ipdfromkm: reconstruct individual patient data from published Kaplan-Meier survival curves. BMC Med Res Methodol. 2021;21:111. 10.1186/s12874-021-01308-8. 34074267 10.1186/s12874-021-01308-8 PMC8168323

[22] Rondeau V, Mazroui Y, Gonzalez JR. frailtypack : An *R* Package for the Analysis of Correlated Survival Data with Frailty Models Using Penalized Likelihood Estimation or Parametrical Estimation. J Stat Softw. 2012;47. 10.18637/jss.v047.i04.

[23] Kassambara A, Kosinski M, Biecek P. survminer: Drawing Survival Curves using “ggplot2.” 2016:0.5.0. 10.32614/CRAN.package.survminer.

[24] Therneau TM. A Package for Survival Analysis in R. 2001:3.8–3. 10.32614/CRAN.package.survival.

[25] Hajime Uno, Lu Tian, Miki Horiguchi, Angel Cronin, Chakib Battioui, James Bell. survRM2: Comparing Restricted Mean Survival Time. 2015:1.0–4. 10.32614/CRAN.package.survRM2.

[26] Duvall JB, Massaad E, Siraj L, Kiapour A, Connolly I, Hadzipasic M, et al. Assessment of spinal metastases surgery risk stratification tools in breast cancer by molecular subtype. Neurosurgery. 2023;92:83–91. 10.1227/neu.0000000000002180. 36305664 10.1227/neu.0000000000002180 PMC10158884

[27] Soto G, Cacho-Diaza B, Bravo-Reynab C, Guerra-Mora J, Ovalles C, Catillo-Rangel C, et al. Prognostic Factors Associated With Overall Survival in Breast Cancer Patients With Metastatic Spinal Disease. CUREUS J Med Sci. 2023;15. 10.7759/cureus.48909. 10.7759/cureus.48909 PMC10725298 38106759

[28] Wang M, Jensen AB, Morgen SS, Wu CS, Sun M, Li H, et al. Survival analysis of breast cancer subtypes in patients with spinal metastases. Spine. 2014;39:1620–7. 10.1097/BRS.0000000000000473. 24979144 10.1097/BRS.0000000000000473

[29] Zhao C, Zhang Z, Zhong N, Fan T, Gao X, Wu Z, et al. Outcomes and prognostic factors for surgically treated patients with breast cancer spine metastases. J Bone Oncol. 2018;12:38–43. 30050751 10.1016/j.jbo.2018.03.003 PMC6058005

[30] Salzer M, Salzer G, Denck H, Brenner H. Operative treatment of “solitary” metastases of the thoracic and lumbar vertebral bodies. Arch Orthop Unfallchir. 1973;75:249–54. 10.1007/BF00416615. 4122288 10.1007/BF00416615

[31] Harrison KM, Muss HB, Ball MR, McWhorter M, Case D. Spinal cord compression in breast cancer. Cancer. 1985;55:2839–44. 10.1002/1097-0142(19850615)55:12<2839::AID-CNCR2820551222>3.0.CO;2-B. 10.1002/1097-0142(19850615)55:12<2839::aid-cncr2820551222>3.0.co;2-b 3995490

[32] Bach F, Larsen BH, Rohde K, Børgesen SE, Gjerris F, Bøge-Rasmussen T, et al. Metastatic spinal cord compression. Occurrence, symptoms, clinical presentations and prognosis in 398 patients with spinal cord compression. Acta Neurochir (Wien). 1990;107:37–43. 10.1007/BF01402610. 2096606 10.1007/BF01402610

[33] Sørensen S, Børgesen SE, Rohde K, Rasmusson B, Bach F, Bøge-Rasmussen T, et al. Metastatic epidural spinal cord compression. Results of treatment and survival Cancer. 1990;65:1502–8. 10.1002/1097-0142(19900401)65:7<1502::AID-CNCR2820650709>3.0.CO;2-D. 10.1002/1097-0142(19900401)65:7<1502::aid-cncr2820650709>3.0.co;2-d 2311062

[34] Boogerd W, van der Sande JJ, Kröger R. Early diagnosis and treatment of spinal epidural metastasis in breast cancer: a prospective study. J Neurol Neurosurg Psychiatry. 1992;55:1188–93. 10.1136/jnnp.55.12.1188. 1479399 10.1136/jnnp.55.12.1188 PMC1015337

[35] Maranzano E, Latini P, Checcaglini F, Perrucci E, Aristei C, Panizza BM, et al. Radiation therapy of spinal cord compression caused by breast cancer: report of a prospective trial. Int J Radiat Oncol Biol Phys. 1992;24:301–6. 10.1016/0360-3016(92)90685-b. 1526868 10.1016/0360-3016(92)90685-b

[36] Hill ME, Richards MA, Gregory WM, Smith P, Rubens RD. Spinal cord compression in breast cancer: a review of 70 cases. Br J Cancer. 1993;68:969–73. 10.1038/bjc.1993.463. 8217611 10.1038/bjc.1993.463 PMC1968743

[37] Tatsui H, Onomura T, Morishita S, Oketa M, Inoue T. Survival rates of patients with metastatic spinal cancer after scintigraphic detection of abnormal radioactive accumulation. Spine. 1996;21:2143–8. 10.1097/00007632-199609150-00017. 8893440 10.1097/00007632-199609150-00017

[38] Rachbauer F, Klestil T, Krismer M, Sterzinger W. Surgical treatment of metastatic disease of the spine. Oncol Res Treat. 1996;19:54–60. 10.1159/000218759.

[39] Shimizu K, Shikata J, Iida H, Iwasaki R, Yoshikawa J, Yamamuro T. Posterior decompression and stabilization for multiple metastatic tumors of the spine. Spine. 1992;17:1400–4. 10.1097/00007632-199211000-00022. 1281341 10.1097/00007632-199211000-00022

[40] Buchelt M, Windhager R, Kiss H, Schneider B, Lack W, Kotz R. [Surgical treatment of spinal metastases]. Z Orthop Ihre Grenzgeb. 1996;134:263–8. 10.1055/s-2008-1039759. 8766130 10.1055/s-2008-1039759

[41] Wagner W, Prott FJ, Rübe C, Willich N. [Radiotherapy of epidural metastases with spinal cord compression]. Strahlenther Onkol Organ Dtsch Rontgengesellschaft Al. 1996;172:604–9. 8975391

[42] Okuyama T, Korenaga D, Tamura S, Maekawa S, Kurose S, Ikeda T, et al. Quality of life following surgery for vertebral metastases from breast cancer. J Surg Oncol. 1999;70:60–3. 10.1002/(SICI)1096-9098(199901)70:1<60::AID-JSO11>3.0.CO;2-U. 10.1002/(sici)1096-9098(199901)70:1<60::aid-jso11>3.0.co;2-u 9989423

[43] Solberg A, Bremnes RM. Metastatic spinal cord compression: diagnostic delay, treatment, and outcome. Anticancer Res. 1999;19:677–84. 10216476

[44] Gokaslan ZL, York JE, Walsh GL, McCutcheon IE, Lang FF, Putnam JB, et al. Transthoracic vertebrectomy for metastatic spinal tumors. J Neurosurg. 1998;89:599–609. 10.3171/jns.1998.89.4.0599. 9761054 10.3171/jns.1998.89.4.0599

[45] Kasai Y, Kawakita E, Uchida A. Clinical profile of long-term survivors of breast or thyroid cancer with metastatic spinal tumours. Int Orthop. 2007;31:171–5. 10.1007/s00264-006-0145-4. 16639592 10.1007/s00264-006-0145-4 PMC2267556

[46] Sakaura H, Hosono N, Mukai Y, Ishii T, Yonenobu K, Yoshikawa H. Outcome of total en bloc spondylectomy for solitary metastasis of the thoracolumbar spine. J Spinal Disord Tech. 2004;17:297–300. 10.1097/01.bsd.0000096269.75373.9b. 15280758 10.1097/01.bsd.0000096269.75373.9b

[47] North RB, LaRocca VR, Schwartz J, North CA, Zahurak M, Davis RF, et al. Surgical management of spinal metastases: analysis of prognostic factors during a 10-year experience. J Neurosurg Spine. 2005;2:564–73. 10.3171/spi.2005.2.5.0564. 15945430 10.3171/spi.2005.2.5.0564

[48] Bilsky MH, Shannon FJ, Sheppard S, Prabhu V, Boland PJ. Diagnosis and management of a metastatic tumor in the atlantoaxial spine. Spine. 2002;27:1062–9. 10.1097/00007632-200205150-00011. 12004173 10.1097/00007632-200205150-00011

[49] Sciubba DM, Gokaslan ZL, Suk I, Suki D, Maldaun MVC, McCutcheon IE, et al. Positive and negative prognostic variables for patients undergoing spine surgery for metastatic breast disease. Eur Spine J. 2007;16:1659–67. 10.1007/s00586-007-0380-4. 17486376 10.1007/s00586-007-0380-4 PMC2078314

[50] Schmidt LA, Pfeiffer P. [Metastatic spinal cord compression syndrome. A retrospective analysis of 297 patients. Ugeskr Laeger. 2006;168:2810–3. 16942703

[51] Ulmar B, Richter M, Cakir B, Muche R, Puhl W, Huch K. The Tokuhashi score: significant predictive value for the life expectancy of patients with breast cancer with spinal metastases. Spine. 2005;30:2222–6. 10.1097/01.brs.0000181055.10977.5b. 16205351 10.1097/01.brs.0000181055.10977.5b

[52] Ampil FL, Baluna R, Burton G, Nanda A. Paraplegia of spinal epidural compression by metastatic breast cancer and urgent radiotherapy-timeliness for naught? J Neurooncol. 2009;95:101–3. 10.1007/s11060-009-9902-8. 19381438 10.1007/s11060-009-9902-8

[53] Gagnon GJ, Henderson FC, Gehan EA, Sanford D, Collins BT, Moulds JC, et al. Cyberknife radiosurgery for breast cancer spine metastases: a matched-pair analysis. Cancer. 2007;110:1796–802. 10.1002/cncr.22977. 17786939 10.1002/cncr.22977

[54] Wibmer C, Leithner A, Hofmann G, Clar H, Kapitan M, Berghold A, et al. Survival analysis of 254 patients after manifestation of spinal metastases: evaluation of seven preoperative scoring systems. Spine. 2011;36:1977–86. 10.1097/BRS.0b013e3182011f84. 21304424 10.1097/BRS.0b013e3182011f84

[55] Ampil F, Caldito G, Thibodeaux J, Sangster G, Baluna R. Radiotherapy for cervical spine metastases in breast cancer patients. Eur J Orthop Surg Traumatol. 2010;20:527–31. 10.1007/s00590-010-0611-y.

[56] Pessina F, Navarria P, Riva M, Franceschini D, Nibali MC, Fornari M, et al. Long-Term Follow-Up of Patients with Metastatic Epidural Spinal Cord Compression from Breast Cancer Treated with Surgery Followed by Radiotherapy. World Neurosurg. 2018;110:e281–6. 10.1016/j.wneu.2017.10.156. 29113903 10.1016/j.wneu.2017.10.156

[57] Switlyk MD, Kongsgaard U, Skjeldal S, Hald JK, Hole KH, Knutstad K, et al. Prognostic factors in patients with symptomatic spinal metastases and normal neurological function. Clin Oncol. 2015;27:213–21. 10.1016/j.clon.2015.01.002. 10.1016/j.clon.2015.01.002 25624156

[58] Tancioni F, Navarria P, Mancosu P, Pedrazzoli P, Morenghi E, Santoro A, et al. Surgery followed by radiotherapy for the treatment of metastatic epidural spinal cord compression from breast cancer. Spine. 2011;36:E1352-1359. 10.1097/BRS.0b013e318207a222. 21358472 10.1097/BRS.0b013e318207a222

[59] Walcott BP, Cvetanovich GL, Barnard ZR, Nahed BV, Kahle KT, Curry WT. Surgical treatment and outcomes of metastatic breast cancer to the spine. J Clin Neurosci. 2011;18:1336–9. 10.1016/j.jocn.2011.02.020. 21782449 10.1016/j.jocn.2011.02.020

[60] Weber A, Bartscht T, Karstens JH, Schild SE, Rades D. Breast cancer patients with metastatic spinal cord compression. Number of extraspinal organs involved by metastases influences survival. Strahlenther Onkol. 2014;190:283–6. 10.1007/s00066-013-0473-4. 24264465 10.1007/s00066-013-0473-4

[61] Rades D, Douglas S, Schild SE. A validated survival score for breast cancer patients with metastatic spinal cord compression. Strahlenther Onkol. 2013;189:41–6. 10.1007/s00066-012-0230-0. 23138773 10.1007/s00066-012-0230-0

[62] Zadnik PL, Hwang L, Ju DG, Groves ML, Sui J, Yurter A, et al. Prolonged survival following aggressive treatment for metastatic breast cancer in the spine. Clin Exp Metastasis. 2014;31:47–55. 10.1007/s10585-013-9608-3. 23999761 10.1007/s10585-013-9608-3

[63] Zakaria HM, Massie L, Basheer A, Boyce-Fappiano D, Elibe E, Schultz L, et al. Application of morphometrics as a predictor for survival in female patients with breast cancer spinal metastasis: a retrospective cohort study. Spine J. 2018;18:1798–803. 10.1016/j.spinee.2018.03.007. 29550605 10.1016/j.spinee.2018.03.007

[64] Sohn S, Kim J, Chung CK, Lee NR, Park E, Chang U-K, et al. A nationwide epidemiological study of newly diagnosed spine metastasis in the adult Korean population. Spine J. 2016;16:937–45. 10.1016/j.spinee.2016.03.006. 26972626 10.1016/j.spinee.2016.03.006

[65] Oliveira M, Rotta JM, Botelho RV. Survival analysis in patients with metastatic spinal disease: the influence of surgery, histology, clinical and neurologic status. Arq Neuropsiquiatr. 2015;73:330–5. 10.1590/0004-282X20150003. 25992524 10.1590/0004-282X20150003

[66] Azad TD, Esparza R, Chaudhary N, Chang SD. Stereotactic radiosurgery for metastasis to the craniovertebral junction preserves spine stability and offers symptomatic relief. J Neurosurg Spine. 2016;24:241–7. 10.3171/2015.6.SPINE15190. 26516666 10.3171/2015.6.SPINE15190

[67] Telera S, Caroli F, Raus L, Pompili A, Carosi MA, Di Santo M, et al. Spine surgery in patients with metastatic breast cancer: a retrospective analysis. World Neurosurg. 2016;90:133–46. 10.1016/j.wneu.2016.02.065. 26906893 10.1016/j.wneu.2016.02.065

[68] Lee SH, Kwon W-K, Ham CH, Na JH, Kim JH, Park Y-K, et al. Postoperative survival after lumbar instrumented surgery for metastatic spinal tumors: a nationwide population-based cohort analysis. Ir J Med Sci. 2024;193:51–6. 10.1007/s11845-023-03459-7. 37450256 10.1007/s11845-023-03459-7

[69] Huang W, Wei H, Cai W, Xu W, Yang X, Liu T, et al. Total En Bloc Spondylectomy for Solitary Metastatic Tumors of the Fourth Lumbar Spine in a Posterior-Only Approach. World Neurosurg. 2018;120:e8-16. 10.1016/j.wneu.2018.06.251. 29990608 10.1016/j.wneu.2018.06.251

[70] Bernard V, Bishop AJ, Allen PK, Amini B, Wang XA, Li J, et al. Heterogeneity in treatment response of spine metastases to spine stereotactic radiosurgery within “radiosensitive” subtypes. Int J Radiat Oncol Biol Phys. 2017;99:1207–15. 10.1016/j.ijrobp.2017.08.028. 29029886 10.1016/j.ijrobp.2017.08.028

[71] McCabe FJ, Jadaan MM, Byrne F, Devitt AT, McCabe JP. Spinal metastasis: the rise of minimally invasive surgery. Surg J R Coll Surg Edinb Irel. 2022;20:328–33. 10.1016/j.surge.2021.08.007. 10.1016/j.surge.2021.08.007 34563452

[72] Rothrock RJ, Barzilai O, Reiner AS, Lis E, Schmitt AM, Higginson DS, et al. Survival trends after surgery for spinal metastatic tumors: 20-year cancer center experience. Neurosurgery. 2021;88:402–12. 10.1093/neuros/nyaa380. 32970144 10.1093/neuros/nyaa380 PMC7803433

[73] Terzi S, Trentin F, Carretta E, Pipola V, Ghermandi R, Barbanti Bròdano G, et al. Breast cancer spinal metastases: prognostic factors affecting survival after surgery. A retrospective study. J Clin Neurosci. 2020;78:73–8. 10.1016/j.jocn.2020.06.010. 32600973 10.1016/j.jocn.2020.06.010

[74] Kato S, Demura S, Murakami H, Shinmura K, Yokogawa N, Annen R, et al. Medium to long-term clinical outcomes of spinal metastasectomy. Cancers. 2022;14:2852. 10.3390/cancers14122852. 35740517 10.3390/cancers14122852 PMC9221216

[75] Chan KS, Shah PV, Shlobin NA, Roumeliotis AG, Thirunavu VM, Larkin CJ, et al. Neurologic, functional, and survival outcomes following surgical management of metastatic breast cancer to the spine. Clin Neurol Neurosurg. 2022;220:107360. 10.1016/j.clineuro.2022.107360. 35868202 10.1016/j.clineuro.2022.107360

[76] Rabah NM, Jarmula J, Hamza O, Khan HA, Chakravarthy V, Habboub G, et al. Metastatic breast cancer to the spine: incidence of somatic gene alterations and association of targeted therapies with overall survival. Neurosurgery. 2023;92:1183–91. 10.1227/neu.0000000000002348. 36735514 10.1227/neu.0000000000002348

[77] Knapp B, Govindan A, Patel SS, Pepin K, Wu N, Devarakonda S, et al. Outcomes in patients with spinal metastases managed with surgical intervention. Cancers. 2024;16:438. 10.3390/cancers16020438. 38275879 10.3390/cancers16020438 PMC10813971

[78] Ciérvide R, Hernando O, López M, Montero Á, Zucca D, Sánchez E, et al. Stereotactic body radiation therapy (SBRT) for spinal metastases: 12 years of a single center experience. Clin Transl Oncol Off Publ Fed Span Oncol Soc Natl Cancer Inst Mex. 2023;25:3395–404. 10.1007/s12094-023-03188-4. 10.1007/s12094-023-03188-4 37058207

[79] Taori S, Adida S, Tang A, Rajan A, Sefcik RK, Burton SA, et al. The role of spine stereotactic radiosurgery for patients with breast cancer metastases. J Neurooncol. 2024;167:257–66. 10.1007/s11060-024-04599-1. 38355870 10.1007/s11060-024-04599-1

[80] Rugo HS, Bardia A, Marmé F, Cortés J, Schmid P, Loirat D, et al. Overall survival with sacituzumab govitecan in hormone receptor-positive and human epidermal growth factor receptor 2-negative metastatic breast cancer (TROPiCS-02): a randomised, open-label, multicentre, phase 3 trial. Lancet. 2023;402:1423–33. 10.1016/S0140-6736(23)01245-X. 37633306 10.1016/S0140-6736(23)01245-X

[81] Modi S, Saura C, Yamashita T, Park YH, Kim S-B, Tamura K, et al. Trastuzumab deruxtecan in previously treated HER2-positive breast cancer. N Engl J Med. 2020;382:610–21. 10.1056/NEJMoa1914510. 31825192 10.1056/NEJMoa1914510 PMC7458671

[82] Gerratana L, Fanotto V, Bonotto M, Bolzonello S, Minisini AM, Fasola G, et al. Pattern of metastasis and outcome in patients with breast cancer. Clin Exp Metastasis. 2015;32:125–33. 10.1007/s10585-015-9697-2. 25630269 10.1007/s10585-015-9697-2

