# Supplementary material for: The influence of immunohistochemistry-based subtypes on overall survival in breast cancer spine metastases: a systematic review and meta-analysis
Source: BMC Med. 2026 Feb 21;24:179. doi: 10.1186/s12916-026-04715-0 (PMC13032407; doi:10.1186/s12916-026-04715-0)
Supplement: Supplementary file 1 — Additional file 1: Search Strategy for studies included in this review. [file 12916_2026_4715_MOESM1_ESM.pdf]

Additional file 1. Search strategy for studies included in this review

The following search strategy, using a combination of MeSH terms and free text terms, was used for MEDLINE, Embase, Web of Sciences, and Google Scholar searches.

MEDLINE

Safari Version 17.6 (17618.3.11.11.7, 17618)

	15-Aug-2024	Results
1	Breast Neoplasms/	343932
2	Spinal Cord Compression/	12029
3	Spinal Neoplasms/	15454
4	("metastatic spinal cord compression*" or "metastatic epidural spinal cord compression*" or "secondary spine tumor*" or "spin* metasta*" or "metastases to the spine").ab,ti.	907
5	2 or 3 or 4	25966
6	1 and 5	652
7	limit 6 to humans	647

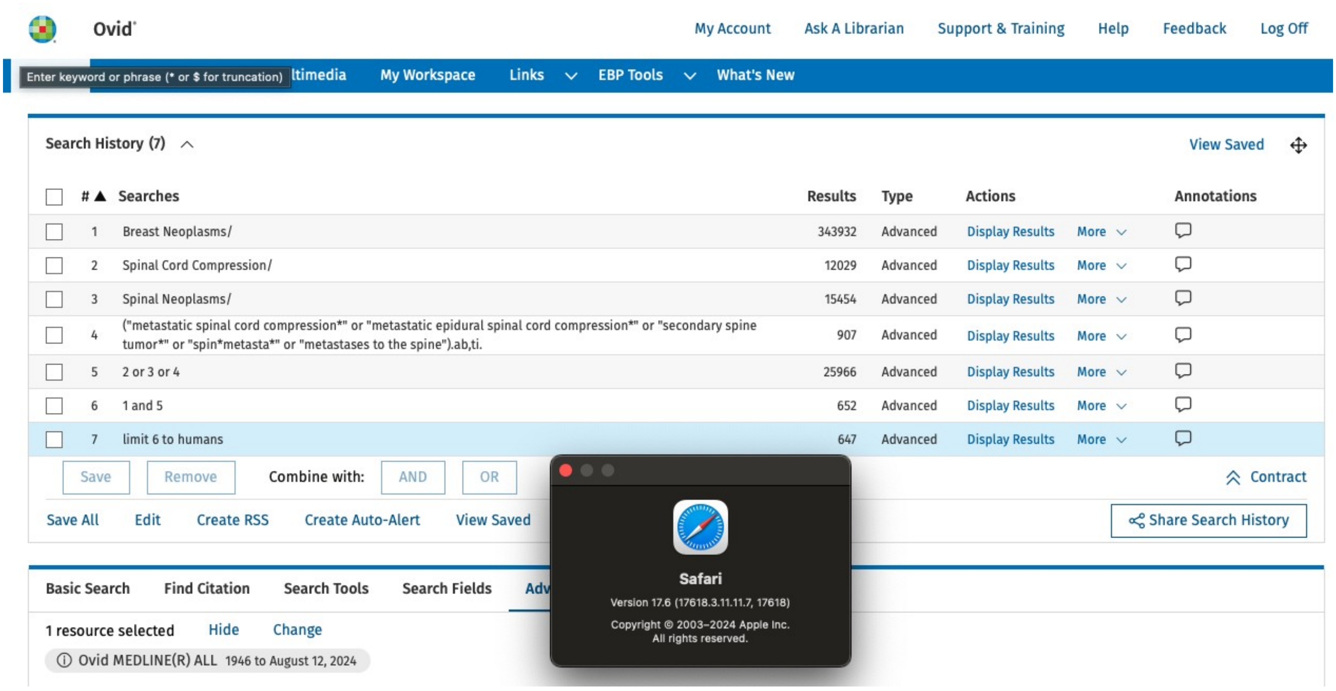

Embase
Safari Version 17.6 (17618.3.11.11.7, 17618)

	15-Aug-2024	Results
1	'breast cancer'	687,869
2	'spinal cord compression'	22,630
3	'spinal cord metastasis'	4,284
4	‘metastatic spinal cord compression'	689
5	‘lumbar spinal canal stenosis’	302
6	'spine tumor'	7,092
7	'spin* metasta*' OR 'metastases to the spine':ab,ti	10,032
8	#2 OR #3 OR #4 OR #5 OR #6 OR #7	38,355
9	#1 AND #8	2,145
10	#1 AND #8 AND [embase]/lim AND [clinical study]/lim AND [humans]/lim	1,425

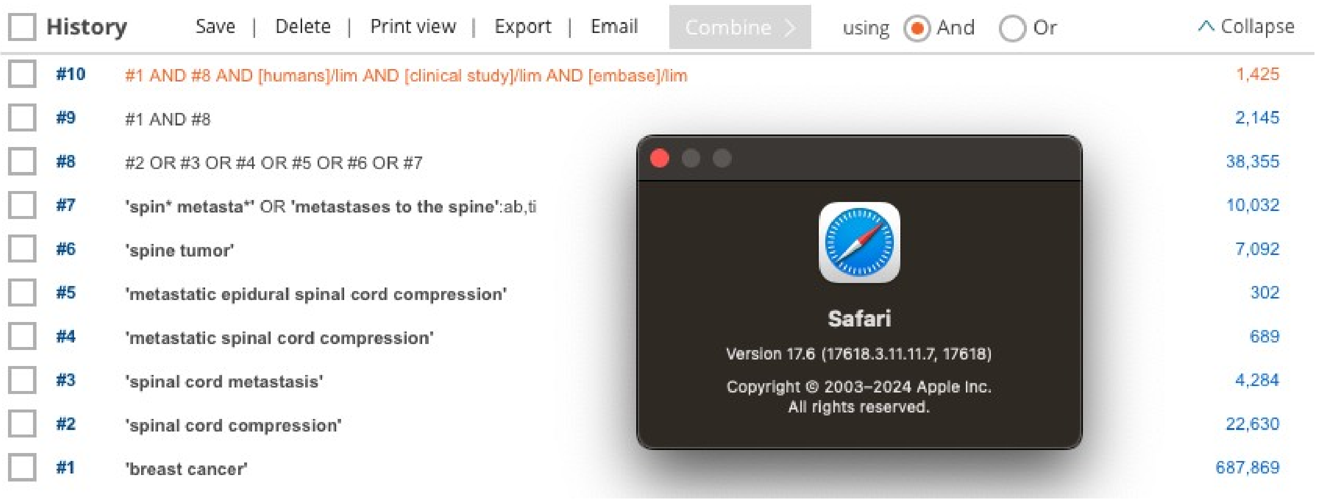

Web of Science
Safari Version 17.6 (17618.3.11.11.7, 17618)

16-Aug-2024,	Results
1. TS = (metastatic breast cancer)	73,465
2. TS = (spinal metastasis) OR TS =(spine)	197,396
3. #1 AND #2	801

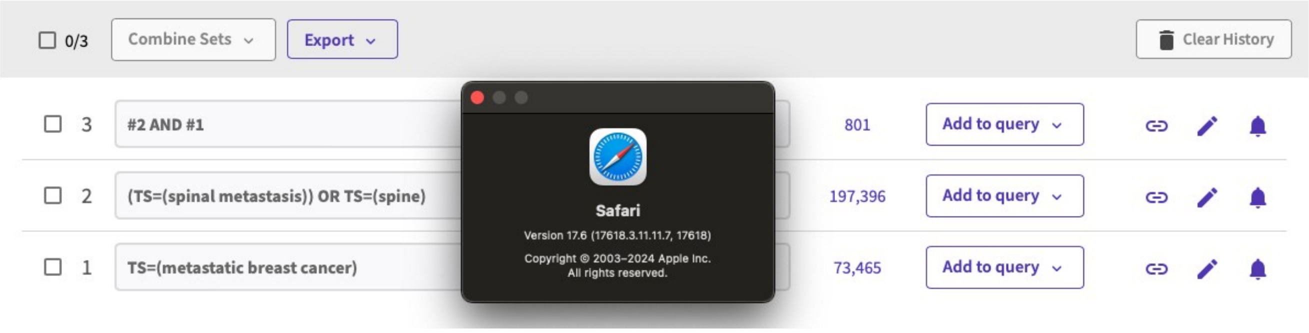

Google Scholar
Safari Version 17.6 (17618.3.11.11.7, 17618)
16-Aug-2024

Related articles of:

Walcott BP, Cvetanovich GL, Barnard ZR, Nahed BV, Kahle KT, Curry WT. Surgical treatment and outcomes of metastatic breast cancer to the spine. J Clin Neurosci. 2011 Oct;18(10):1336-9
