# Supplementary material for: The influence of immunohistochemistry-based subtypes on overall survival in breast cancer spine metastases: a systematic review and meta-analysis
Source: BMC Med. 2026 Feb 21;24:179. doi: 10.1186/s12916-026-04715-0 (PMC13032407; doi:10.1186/s12916-026-04715-0)
Supplement: Supplementary file 2 — Additional file 2: Data extraction form. [file 12916_2026_4715_MOESM2_ESM.pdf]

Additional file 2. Data extraction form

Data Extraction Form 1.0

Study ID:______________________

Extracted by: □ Yun-Heng Li □ Chai Chung Liang □ Ting-Li Shen

Citation:____
____
____
____

Population:

□ Compatible with breast cancer with spinal metastasis
□ Not-compatible

Outcome

Insert survival curve/cummulative events curve for data extraction

Subgroup/Moderator analysis

Mean age:_______ (SD:_____)

GET IT ON Google Play
Download on the App Store
怡仁綜合醫院 Yeezen General Hospital
326桃園市楊梅區楊新北路321巷30號
03-4855566
怡仁綜合醫院粉絲團
民眾意見反映電子信箱
