# Supplementary material for: The influence of immunohistochemistry-based subtypes on overall survival in breast cancer spine metastases: a systematic review and meta-analysis
Source: BMC Med. 2026 Feb 21;24:179. doi: 10.1186/s12916-026-04715-0 (PMC13032407; doi:10.1186/s12916-026-04715-0)
Supplement: Supplementary file 3 — Additional file 3: GRADE definitions and judgment process. [file 12916_2026_4715_MOESM3_ESM.pdf]

Additional file 3. GRADE definitions and judgment process

GRADE is an abbreviation of "Grades of Recommendation, Assessment, Development, and Evaluation.'' The complete formal description is in the publication about GRADE (Guyatt et al.).

Our meta-analysis summarizes single-arm survival functions, whereas the original GRADE framework is designed for comparisons between two groups. Therefore, we adapted the GRADE criteria by narrowing its scope and referred to the paper by Murad et al., *'Rating the certainty in evidence in the absence of a single estimate of effect,'* as well as GRADE Guideline 28 to guide our judgment process.[16, 18]

Domains of GRADE	Definition
Methodological limitation (Risk of bias/study limitations)	To check for problems in the design or conduct of a study, we narrowed the original criteria to judge the selection of exposed and unexposed from different populations. We therefore assess the enrollment criteria employed in these published studies for selection bias, which could constrain the external validity when applying these results to the target population implied by our study.
Inconsistency	Judge inconsistency by evaluating the consistency of the direction and primarily the difference in the magnitude of effects across studies. Widely differing estimates of the effects indicate inconsistency. Also considers the unexplained heterogeneity of results.
Indirectness	Revisit population, intervention, comparator, and outcomes (PICO) to judge whether the effect summary has a direct relationship with it.
Imprecision	Studies with small number of patients and low event rates are likely to be not accurate – imprecise. This can be evident when there is a wide confidence interval around the effect estimate.
Publication bias	This domain focused on whether there is a selective publication of studies that may cause systematic under-estimation or an over-estimation of the underlying treatment effect.

The meaning of the outcome of GRADE judgment

High certainty:	We are very confident that the variation in risk associated with the prognostic factor (probability of future events in those with/ without the prognostic factor) lies close to that of the estimate
Moderate certainty:	We are moderately confident that the variation in risk associated with the prognostic factor (probability of future events in those with/without the prognostic factor) is likely to be close to the estimate, but there is a possibility that it is substantially different
Low certainty	Our certainty in the estimate is limited: the variation in risk associated with the prognostic factor (probability of future events in those with/without the prognostic factor) may be substantially different from

	the estimate
Very low certainty:	We have very little certainty in the estimate: the variation in risk associated with the prognostic factor (probability of future events in those with/without the prognostic factor) is likely to be substantially different from the estimate

Risk of Bias Assessments of the Included Studies Using QUIPS tool [17] in the outcome of **HR+, HER2+, and Triple Negative Breast Cancer**

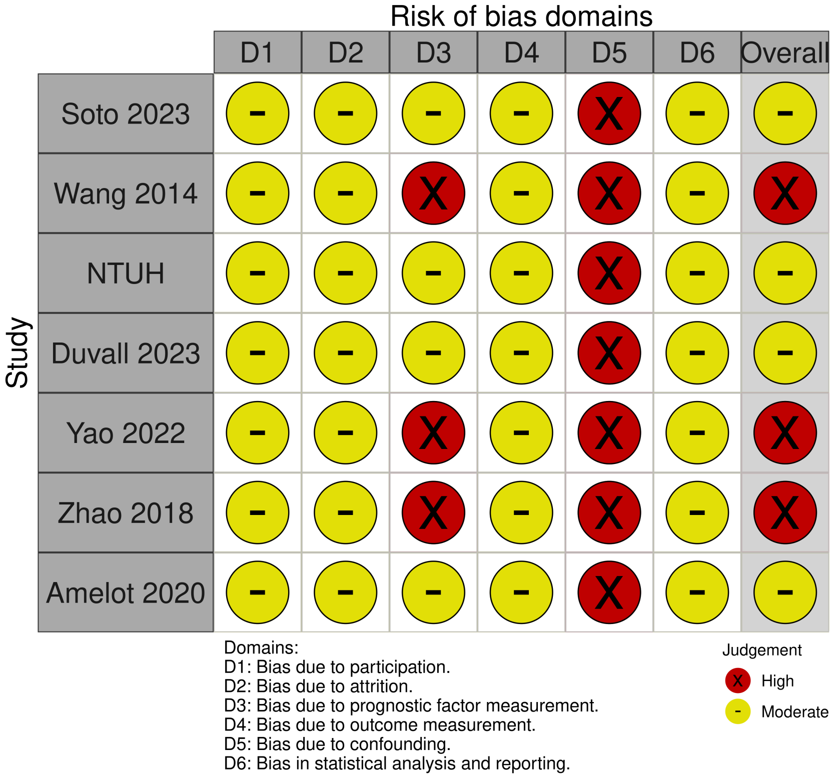

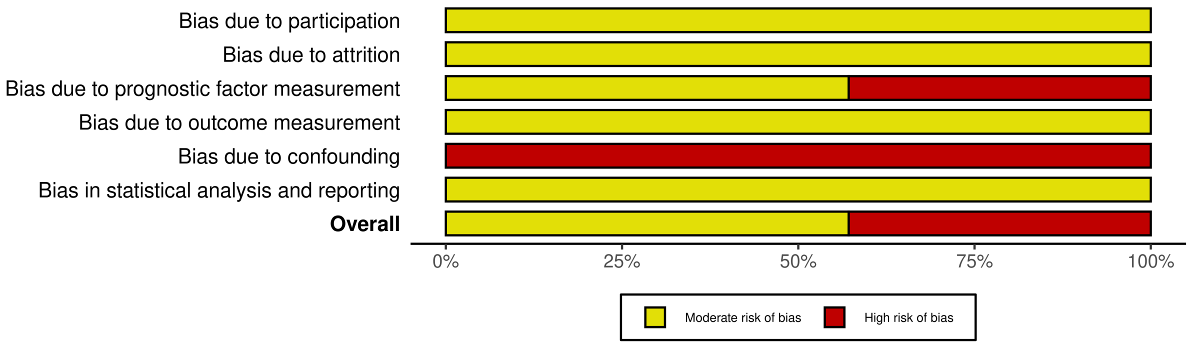

Detailed Processes of GRADE on each outcomes: **HR+, HER2+, and Triple Negative Breast Cancer**

GRADE domain	Interpretation and consideration	Concerns about certainty domains
Methodological limitations of the studies	Three of the seven included studies (Amelot 2020, Soto 2023, and Wang 2014) exhibited potential biases that may introduce uncertainty in their respective findings. QUIPS assessment outcome indicates high risk of bias.	Serious
Inconsistency	Although the estimated frailty parameter (θ) did not reach statistical significance for quantifying inter-study heterogeneity, substantial clinical heterogeneity persists. The overrepresentation of surgically managed patients from high-resource tertiary referral centers introduces three critical selection biases: (1) inherent selection of optimal surgical candidates, (2) socioeconomic stratification affecting access to advanced diagnostics and therapies, and (3) institutional variability in palliative care protocols. Consequently, we graded the inconsistency as serious in accordance with GRADE methodology.	Serious
Indirectness	Given the consistent reporting standards for molecular profiling across all included studies, we determined the risk of indirectness to be negligible according to GRADE criteria.	Not serious
Imprecision	The relatively narrow confidence intervals observed for the survival function estimates supported our GRADE assessment of 'not serious' in the domain of imprecision.	Not serious, borderline
Publication bias	Although our analysis incorporated data from 61 studies (n=4,465), only 7 studies (n=672) provided extractable molecular profiling data, representing merely 15% of the total cohort. The geographic concentration of these molecularly-characterized studies (predominantly from China, Denmark, France, Mexico, Taiwan, and the United States) raises substantial concerns regarding publication bias. This limited representation may compromise the generalizability of the survival estimates to under-represented regions including ASEAN nations, South America, the Middle East, and Africa. Consequently, we rated the evidence as having serious potential for regional estimation bias in the GRADE assessment.	Serious

**GRADE criteria**	**Rating** (highlight one)	**Footnotes** (explain reasons for down- or upgrading)	**Certainty of Evidence** (highlight one)
**Outcome:**			
**Study design**	According to GRADE guideline 28, for questions of prognosis, a body of observational evidence begins as high certainty in the evidence.		
**Methodological Limitation** (use GRADE limitation guideline 4)	No<br>serious (-1)<br>very serious (-2)	QUIPS outcome is serious	
**Inconsistency**	No<br>serious (-1)<br>very serious (-2)	substantial clinical heterogeneity	⦿⦿⦿⦿ High
**Indirectness**	No<br>serious (-1)<br>very serious (-2)	consistent reporting standards for molecular profiling	⦿⦿⦿○ Moderate<br>⦿⦿○○ Low
**Imprecision**	No<br>serious (-1)<br>very serious (-2)	relatively narrow confidence intervals for the survival estimates	⦿○○○ Very Low
**Publication Bias**	Undetected<br>Strongly suspected (-1)	few countries publishes the data	
**Other (upgrading factors, highlight all that apply)**	Large effect (+1 or +2) Dose response (+1 or +2)<br>No Plausible confounding (+1 or +2)	We have not identified these factors that warrant an upgrade.	
