# Supplementary material for: The influence of immunohistochemistry-based subtypes on overall survival in breast cancer spine metastases: a systematic review and meta-analysis
Source: BMC Med. 2026 Feb 21;24:179. doi: 10.1186/s12916-026-04715-0 (PMC13032407; doi:10.1186/s12916-026-04715-0)
Supplement: Supplementary file 4 — Additional file 4: Reasons for excluding 34 eligible studies. [file 12916_2026_4715_MOESM4_ESM.pdf]

Additional file 4. Reasons for excluding 34 eligible studies

	Reasons	Title of Article	Source publication	First author
1	Published data does not permit extraction into useable data according to protocol	Trends in survival after surgery for breast cancer metastatic to the brain and spinal column in medicare patients: a population-based analysis.	Neurosurgery. 2011 Mar;68(3):705–713. doi: 10.1227/NEU.0b013e31820773b2	Kevin S Cahill
2	Cohort from the same institute as DOI: 10.1007/s00066-014-0651-z	Prognostic factors for survival of women with unstable spinal bone metastases from breast cancer.	Radiat Oncol. 2015 Jul 15:10:144. doi: 10.1186/s13014-015-0458-9.	Robert Foerster
3	The study's 12-year observation period yielded exceptionally long-term survival data, with follow-up extending to 25 years in some cases.	Stability of spinal bone metastases in breast cancer after radiotherapy: a retrospective analysis of 157 cases	Strahlenther Onkol. 2014 Sep;190(9):792-7. doi: 10.1007/s00066-014-0651-z.	Ingmar Schlampp
4	The patient survival is not compatible with the definition of overall survival	Asymptomatic Construct Failure after Metastatic Spine Tumor Surgery: A New Entity or a Continuum with Symptomatic Failure?	Asian Spine J. 2021 Oct;15(5):636-649. doi: 10.31616/asj.2020.0167. Epub 2020 Oct 29.	Naresh Kumar
5	Part of the spine metastasis group is within bone metastasis	Incidence and prognostic factors of Japanese breast cancer patients with bone metastasis.	J Orthop Sci. 2006 Jan;11(1):13-9. doi: 10.1007/s00776-005-0966-9.	Hiroyuki Oka
6	Cohort from University Hospital Schleswig-Holstein, Lübeck, Mayo Clinic, Scottsdale, already included	A new instrument for estimation of survival in elderly patients irradiated for metastatic spinal cord compression from breast cancer.	Radiat Oncol . 2015 Aug 19:10:173. doi: 10.1186/s13014-015-0483-8.	Dirk Rades
7	Cohort from University Hospital Schleswig-Holstein, Lübeck, Mayo Clinic, Scottsdale, already included	Outcomes After Radiotherapy Alone for Metastatic Spinal Cord Compression in Patients with Oligo-metastatic Breast Cancer.	Anticancer Res. 2018 Dec;38(12):6897-6903. doi: 10.21873/anticanres.13066.	Dirk Rades
8	Cohort from IRCCS Istituto Clinico Humanitas, Rozzano, already included	Multimodal approach to the management of metastatic epidural spinal cord compression (MESCC) due to solid tumors	Int J Radiat Oncol Biol Phys. 2010 Dec 1;78(5):1467-73. doi: 10.1016/j.ijrobp.2009.09.038.	Flavio Tancioni
9	Cohort University of Ulm, already included	Surgical treatment and prognosis factors in spinal metastases of breast cancer	Z Orthop Ihre Grenzgeb . 2005 Mar-Apr;143(2):186-94. doi: 10.1055/s-2005-836512.	B Ulmar
10	Cohort University of Ulm, already included	The prognostic influence of primary tumour and region of the affected spinal segment in 217 surgical patients with spinal metastases of different entities	Z Orthop Ihre Grenzgeb . 2007 Jan-Feb;145(1):31-8. doi: 10.1055/s-2007-960506.	B Ulmar
11	Contains 14 breast cancer patients but the data cannot	Surgical treatment of spinal cord compression from epidural	Journal of Clinical Oncology, Volume	Sundaresan, Narayan

	be extracted.	metastasis.	13, Number 9 https://doi.org/10.1200/JCO.1995.13.9.2330	
12	Contains 10 breast cancer patients but the data cannot be extracted.	Metastatische epidurale Spinalkanalkompression: Prognostische Faktoren und Ergebnisse der Strahlentherapie	Strahlenther Onkol 177, 676–679 (2001). https://doi.org/10.1007/PL00002383	Lövey, G
13	Contains 335 breast cancer patients but the data cannot be extracted.	Prognostic factors predicting functional outcomes, recurrence-free survival, and overall survival after radiotherapy for metastatic spinal cord compression in breast cancer patients	International Journal of Radiation Oncology, Biology, Physics, Volume 64, Issue 1, 182 - 188	Rades, Dirk
14	Contains 27 breast cancer patients but the data cannot be extracted.	Outcomes and survival of spinal metastasis with epidural compression	Journal of Craniovertebral Junction and Spine 12(3):p 287-293, Jul–Sep 2021.	Candido, Priscila Barile Marchi
15	Contains 44 breast cancer patients but the data cannot be extracted.	Assessment of the posterior approach for surgical treatment of spinal metastatic breast cancer	Acta Ortopedica Brasileira	Pontes, Mariana Demétrio de Sousa
16	Contains 87 breast cancer patients but the data cannot be extracted.	Surgical treatment strategies and outcome in patients with breast cancer metastatic to the spine: a review of 87 patients	Eur Spine J (2007) 16:1179–1192 DOI 10.1007/s00586-007-0357-3	Joseph A. Shehadi
17	Contains 25 breast cancer patients but the data cannot be extracted.	Early Treatment of Spinal Epidural Compression in Breast Cancer Patients without Neurological Deficits Has the Potential for Improved Outcome	The Breast Journal. Vol. 21 Issue 6, pp. 688–690, 2015.	Ampil, Federico
18	Does not contain data despite PICO compatible	Diagnosis and surgical management of breast cancer metastatic to the spine	World J Clin Oncol 2014 August 10; 5(3): 263-271	Derek G Ju
19	Contains 185 breast cancer patients but the data cannot be extracted.	Evaluation of Prognostic Factors and Proposed Changes to the Modified Tokuhashi Score in Patients With Spinal Metastases From Breast Cancer	Spine. Vol. 43 Issue 7, pp. 512–519, 2018.	Tan, Kimberly-Anne
20	Contains 105 breast cancer patients but the data cannot be extracted.	Epidural extension of vertebral metastases is not an independent predictor of prognosis after therapy in breast cancer patients	Journal of Neuro-Oncology Vol. 125 Issue 2, pp. 437–438, 2015	Ampil, Federico
21	Part of the spine metastasis group is within bone metastasis	Prognostic factors for skeletal complications from metastatic bone disease in breast cancer	Breast Cancer Research and Treatment. Vol. 123 Issue 3, pp. 767–779, 2010.	Brown, Janet E.
22	Contains 2 breast cancer patients but the data cannot be extracted.	Symptomatic Spinal Cord Necrosis After Irradiation for Vertebral Metastatic Breast Cancer	Journal of Clinical Oncology (JCO). Vol. 29 Issue 3, pp. e53–e56, 2011.	Pompili, Alfredo
23	Contains 140 breast cancer patients but the data cannot be extracted.	Spinal Metastases in Breast Cancer: Single Center Experience	World Neurosurg. (2014) 82, 6:1344-1350.	Emilie Chan-Seng
24	Contains breast cancer patient but the data cannot be extracted.	Current trends in incidence, characteristics, and surgical management of metastatic breast	Journal of Clinical Neuroscience, Volume 91, 99 - 104	Keyan A. Peterson

		cancer to the spine: A National Inpatient Sample analysis from 2005 to 2014		
25	Part of the spine metastasis group is within bone metastasis	Efficacy of ibandronate for the treatment of skeletal events in patients with metastatic breast cancer	European Journal of Cancer Car. Vol. 18 Issue 6, pp. 653–656, 2009.	HERAS, P.
26	Contains 43 breast cancer patients but the data cannot be extracted.	Percutaneous Vertebroplasty Combined with Zoledronic Acid for the Treatment of Painful Osteolytic Spinal Metastases in Patients with Breast Cancer	Journal of Vascular and Interventional Radiology Vol. 24 Issue 12, pp. 1861–1867, 2013.	Zhang, Jianjun
27	Contains 56 breast cancer patients but the data cannot be extracted.	Clinical outcome in metastatic spinal cord compression. A prospective study of 153 patients	Acta Neurol Scand . 1996 Oct;94(4):269-75. doi: 10.1111/j.1600-0404.1996.tb07064.x .	S. Helweg-Larsen
28	Part of the spine metastasis group is within bone metastasis	Prognostic Factors for Bone Survival and Functional Outcomes in Patients With Breast Cancer Spine Metastases	Technology in Cancer Research & Treatment Vol. 21, 2022	Qiao, Rui-qi
29	Contains 26 breast cancer patients but the data cannot be extracted.	Vertebral compression fractures in patients presenting with metastatic epidural spinal cord compression	Neurosurgery 2009 Aug;65(2):267-74	Kaisorn L Chaichana
30	Contains 44 breast cancer patients but the data cannot be extracted.	Assessment of the posterior approach for surgical treatment of spinal metastatic breast cancer	Acta Ortop Bras . 2020 Jan-Feb;28(1):22-25. doi: 10.1590/1413-785220202801223529.	Mariana Demétrio de Sousa Pontes
31	Contains 11 breast cancer patients but the data cannot be extracted.	Impact of decompressive laminectomy on the functional outcome of patients with metastatic spinal cord compression and neurological impairment	Clinical & Experimental Metastasis Vol. 37 Issue 2, pp. 377–390, 2020.	Younsi, Alexander
32	Contains 15 breast cancer patients but the data cannot be extracted.	Does the Pathologic Fracture Predict Severe Paralysis in Patients with Metastatic Epidural Spinal Cord Compression (MESCC)?—A Retrospective, Single-Center Cohort Analysis	Journal of Clinical Medicine. Vol. 12 Issue 3, p. 1167, 2023.	Klein, Lukas
33	Breast cancer grouped as part of “favourable pathology” and cannot be extracted	A Personalized Medicine Approach for the Management of Spinal Metastases with Cord Compression: Development of a Novel Clinical Prediction Model for Postoperative Survival and Quality of Life	World Neurosurgery Volume 140, August 2020, Pages 654-663.e13	Anick Nater
34	Contains 462 breast cancer patients but the data cannot be extracted.	Survival after surgery for spinal metastases: a population-based study	Can J Surg 2022 August 4; 65(4). doi: 10.1503/cjs.000921	Kunal Bhanot
