# Supplementary material for: The influence of immunohistochemistry-based subtypes on overall survival in breast cancer spine metastases: a systematic review and meta-analysis
Source: BMC Med. 2026 Feb 21;24:179. doi: 10.1186/s12916-026-04715-0 (PMC13032407; doi:10.1186/s12916-026-04715-0)
Supplement: Supplementary file 5 — Additional file 5: Study selection and inter-assessor agreement. [file 12916_2026_4715_MOESM5_ESM.pdf]

Additional file 5. Study selection and inter-assessor agreement

1st Reviewer: Chung Liang Chai
2nd Reviewer: Yun-Heng Li
3rd Reviewer: Fon-Yih Tsuang

Each reviewer independently assessed the studies for inclusion or exclusion. Subsequently, their decisions were compared to identify any discrepancies. Chai CL then reported the inter-assessor reliability, using Cohen’s kappa statistic, to Fon-Yih Tsuang. All disagreements and accuracy concerns were reviewed, resolved, and overseen by Fon-Yih Tsuang.

Inter-rater reliability analysis yielded an excellent Cohen's kappa coefficient of 0.91 (95% CI: 0.84-0.98), with 96% observed agreement between raters.

Please see the table below for the discussion.

Cohen’s kappa is 0.91 (96% agreement)

n = 135		Li’s decision	
		Inclusion	Not to include
Chai’s decision	Inclusion	58	2
	Not to include	4	71

Only Chai want to include

Final Decision	Study Name	Discussion Process	Comments from Tsuang
Exclude	Schlampp et al<br>Stability of spinal bone metastases in breast cancer after radiotherapy: a retrospective analysis of 157 cases<br><br>Strahlenther Onkol. 2014 Sep;190(9):792-7.	Li: The study's 12-year observation period yielded exceptionally long-term survival data, with follow-up extending to 25 years in some cases.<br><br>Chai: The study has comparable data as defined in our protocol.	I agree with Li, after rechecking the publication content.
Exclude	Anick Nater et al.<br>A personalized medicine approach for the management of spinal metastases with cord compression<br><br>World Neurosurgery Volume 140, August 2020, Pages 654-663.e13	Li: Breast cancer grouped as part of “favourable pathology” and cannot be extracted<br><br>Chai: The study has comparable data as defined in our protocol.	I agree with Li. After recheck, the data is incompatible with our protocol.

Only Li want to include

Final Decision	Study Name	Discussion Process	Comments from Tsuang
Exclude	Kumar et al.<br>Asymptomatic Construct Failure after Metastatic Spine Tumor Surgery: A New Entity or a Continuum with Symptomatic Failure?<br><br>Asian Spine J. 2021 Oct;15(5):636-649	Chai: The patient survival is not compatible with the definition of overall survival<br><br>Li: The study has comparable data as defined in our protocol.	I agree with Chai, after rechecking the publication content.
Include	Seil Sohn et al.<br>A nationwide epidemiological study of newly diagnosed spine metastasis in the adult Korean population<br><br>Spine J. 2016 Aug;16(8):937-45.	Chai: The data is from Korean Health Insurance Review and Assessment Service database and with duplication with Lee 2024, also from same database.<br><br>Li: The study has comparable data as defined in our protocol.	The compatibility remains uncertain; we may consider including it initially to evaluate its impact on our analysis.
Exclude	Hiroyuki Oka et al.<br>Incidence and prognostic factors of Japanese breast cancer patients with bone metastasis.<br><br>J Orthop Sci. 2006 Jan;11(1):13-9.	Chai: Part of the spine metastasis group is within bone metastasis<br><br>Li: The study has comparable data as defined in our protocol.	I agree with Chai, after rechecking the publication content.
Exclude	Kevin S Cahill et al.<br>Trends in survival after surgery for breast cancer metastatic to the brain and spinal column in medicare patients: a population-based analysis.<br><br>Neurosurgery. 2011 Mar;68(3):705-13	Chai: Published data does not permit extraction into useable data according to protocol<br><br>Li: The study has comparable data as defined in our protocol.	I agree with Chai, after rechecking the publication content.
