# Supplementary material for: The influence of immunohistochemistry-based subtypes on overall survival in breast cancer spine metastases: a systematic review and meta-analysis
Source: BMC Med. 2026 Feb 21;24:179. doi: 10.1186/s12916-026-04715-0 (PMC13032407; doi:10.1186/s12916-026-04715-0)
Supplement: Supplementary file 6 — Additional file 6: Median overall survival: digitized vs. reported estimates. [file 12916_2026_4715_MOESM6_ESM.pdf]

Additional file 6. Median overall survival: digitized vs. reported estimates.

A cross-check of study-level median overall survival (OS) derived from digitized survival curves against the statistics originally reported in text.

Study	Reconstructed median OS months	Reported median OS months
Amelot 2020	42 (95% CI: 33–47)	43.9 (SD 5.7)
Ampil 2009	5 (95% CI: 1–infinite)	5
Ampil 2010	3 (95% CI: 2–17)	3
Azad 2016	81 (95% CI: 2–infinite)	Not reported, individual breast CA survival provided in Table 1 in original publication.
Bach 1990	3.62 (95% CI: 2.53–7.27)	Provided survival curve but did not report median overall survival
Bernard 2017	Did not reach 50% at the end of FU	Did not reach 50% at the end of FU
Bilsky 2002	7.5 (95% CI: 5–infinite)	Not reported, individual breast CA survival provided in Table 1 in original publication.
Boogerd 1992	multiple: 12.13 (95% CI: 3.29–22.1)<br>single:7.16 (95% CI: 3.77–16.2)	multiple: not reported<br>single: not reported
Buchelt 1996	5.84 (95% CI:4.74–37.1)	Mean survival time: 13.9 ± 4.0
Chan 2022	83.7% (95% CI: 73.9–94.7) at 1 year	89.2 % (95% CI: 80.7–98.6) at 1 year
Ciérvide 2023	100% at 3 year	100% at 3 year
Duvall 2023	61.1% (95% CI: 52.1–71.5) at 1 year	57% at 1 year
Gagnon 2007	21 (95% CI: 11.9–59.7)	Not reported, despite KM curve provide
Gokaslan 1998	60% (95% CI: 36.2–99.5) at 1 year	63% at 1 year
Harrison 1985	11 (95% CI: 5.69–13.9)	8.2 (95% CI: 0.8–53.9)
Hill 1993	3.68 (95% CI: 2.04–5.65)	4 (range: 0-56)
Huang 2018	Only 2 survival data available	Not reported, individual breast CA survival provided in Table 3 in original publication.
Kasai 2007	36 (95% CI: 12–infinite)	Not reported, individual breast CA survival provided in Table 1 in original publication.
Kato 2022	66.7% (95% CI: 44.7–99.5) at 5 year	66.7% at 5 year
Knapp 2024	28.5 (95% CI: 17.1–infinite)	28.6 (95% CI: 17.0–29.8)

Lee 2024	26.5 (95% CI: 8.94–41.5)	26.76 ± 21.96
Maranzano 1992	15 (95% CI: 9-21)	13 (range 2–37)
McCabe 2022	65.3% (95% CI: 53.3-80.1) at 1 year	69% at 1 year
North 2005	21.5 (95% CI: 17.3–infinite)	20.4
Okuyama 1999	29 (95% CI: 17–infinite)	Not reported, individual breast CA survival provided in Table 1 in original publication.
Oliveira 2015	26.2 (95% CI: 11.2–infinite)	28
Pessina 2018	47 (95% CI: 33.5–infinite)	47 (95% CI: 33–114)
Rabah 2023	59.4 (95% CI: 50.6–infinite)	Not reported
Rachbauer 1996	15.4 (95% CI: 4.39–infinite)	12.4
Rades 2013	Survival % at 12 months<br>test arm<br>Group A: 3.85 (95% CI: 5.63-26.3)<br>Group B: 23.5 (95% CI: 14.4-38.6)<br>Group C: 59.0 (95% CI: 47.9-72.7)<br>Group D: 90.6 (95% CI: 85.5- 96.0)<br>validate arm<br>Group A: 7.14 (95% CI: 18.8-27.2)<br>Group B: 38.0 (95% CI: 26.7-54.1)<br>Group C: 66.7 (95% CI: 54.6-81.4)<br>Group D: 87.6 (95% CI: 82.1-93.5)	Survival % at 12 months<br>test arm<br>Group A: 4<br>Group B:16<br>Group C:55<br>Group D: 90<br>validate arm<br>Group A: 4<br>Group B: 34<br>Group C: 67<br>Group D: 88
Rothrock 2021	17.1 (95% CI: 12.6–25.3)	16.6 (95% CI: 12–24.5)
Sakaura 2004	13 (95% CI: 7–infinite)	Not reported, individual breast CA survival provided in Table 1 in original publication.
Salzer 1973	12 (95% CI: 11–infinite)	Not reported, individual breast CA survival provided in Table 1 in original publication.
Schmidt 2006	6.9 (95% CI: 4.87–17.5)	Not reported, breast CA survival curve provided in Figure 1 in original publication.
Sciubba 2007	20.1 (95% CI: 17.5–32.6)	21 (95% CI: 16–27)
Shimizu 1992	Not reached 50% survival at the end of follow-up	Not reported, individual breast CA survival provided in Table 1 in original publication.
Sohn 2016	71.2% (95% CI: 68.4–74.1) at 1 year	71.5% at 1 year
Solberg 1999	14.9 (95% CI: 3.86–infinite)	14.8
Sørensen 1990	10.2 (95% CI: 6.78–18.8)	9.2 (range: 0.6-49.3)
Soto 2023	57.88	59.90 ± 4.89

Switlyk 2015	77.3% (95 CI: 65.8–90.7) at 1 year	77% at 1 year
Tancioni 2011	36 (95% CI: 13–infinite)	36 (range: 3–60)
Taori 2024	32 (95% CI: 20–44)	32 (range: 2–183)
Tatsui 1996	75.3% (95% CI: 66.8-84.8) at 1 year	77.7% at 1 year
Telera 2016	50 (95% CI: 36.1–infinite)	50 (95% CI: 35–65)
Terzi 2020	60.6% (95% CI: 48.9–75) at 3 year	61% (95% CI: 47.5–72.1) at 3 year
Ulmar 2005	Group 1 3.06 (95% CI: 0.8–infini.)<br>Group 2 21.61 (95% CI: 11.3–36.3<br>Group 3 27.43 (95% CI: 17.2–86.8)	Group 1 2.9<br>Group 2 14.4<br>Group 3 25.6
Wagner 1996	20.7 (95% CI: 12.4–infinite)	23.3
Walcott 2011	35.1 (95% CI: 29.6–infinite)	33.7
Wang 2014	21.2 (95% CI: 16–26)	21.2 (95% CI: 15.9–25.1)
Weber 2014	1 year survival involvement of extraspinal organ<br>0: 90.2% (95% CI: 81.6–99.8)<br>1: 75.9% (95% CI: 65.3–88.2)<br>2: 48.4% (95% CI: 33.6–69.6)<br>≥3: 15.8% (95% CI: 5.59–44.6)	1 year survival involvement of extraspinal organ<br>0: 86%<br>1: 73%<br>2: 36%<br>≥3: 16%
Wibmer 2011	26.2 (min: 1.1; max: 128.0)	24.2 (min: 1.0; max: 128.4)
Wright 2018	22.8 (95% CI: 20.8–25.2)	Not reported, breast CA survival curve provided in Figure 8 in original publication.
Yao 2022	33.5 (95% CI: 23.7–infinite)	33.6
Zadnik 2014	26.3 (95% CI: 12.8–32.9)	26.8
Zakaria 2018	3.43 (95% CI: 2.62–5.15)	3.4 (95% CI: 2.4–5.2)
Zhao 2018	41.9 (95% CI: 17–infinite)	36.0 (95% CI: 21.3–50.7).

Abbreviations
CA: cancer
CI: confidence interval
FU: follow-up
