# Supplementary material for: The influence of immunohistochemistry-based subtypes on overall survival in breast cancer spine metastases: a systematic review and meta-analysis
Source: BMC Med. 2026 Feb 21;24:179. doi: 10.1186/s12916-026-04715-0 (PMC13032407; doi:10.1186/s12916-026-04715-0)
Supplement: Supplementary file 7 — Additional file 7: Forest plot of the hazard ratio for HER2 +/HR + compared to HER2 +/HR-. [file 12916_2026_4715_MOESM7_ESM.pdf]

Additional file 7. Forest plot of the hazard ratio for HER2+/HR+ compared to HER2+/HR-

Survival outcomes were compared between HER2+/HR+ and HER2+/HR- subtypes in a post hoc subgroup analysis of HER2-positive patients. Study-specific hazard ratios were pooled under a random-effects model. NTUH: National Taiwan University Hospital

Source	HR (95% CI)
Soto 2023	1.42 [0.18; 11.42]
NTUH	0.41 [0.22; 0.76]
Duvall 2023	0.04 [0.00; 0.42]
Zhao 2018	0.77 [0.26; 2.27]
Amelot 2020	0.68 [0.36; 1.28]
Total	0.53 [0.35; 0.80]

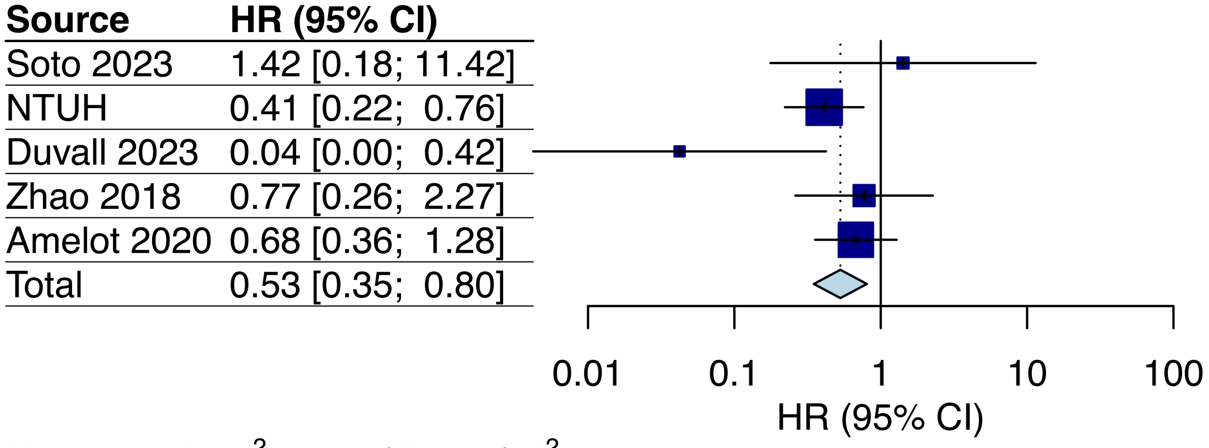

Heterogeneity: $\chi^2_4 = 7.16$ ($P = .13$), $I^2 = 44.1\%$
