# Supplementary material for: The influence of immunohistochemistry-based subtypes on overall survival in breast cancer spine metastases: a systematic review and meta-analysis
Source: BMC Med. 2026 Feb 21;24:179. doi: 10.1186/s12916-026-04715-0 (PMC13032407; doi:10.1186/s12916-026-04715-0)
Supplement: Supplementary file 8 — Additional file 8: Characteristics of included studies for survival changes over time. [file 12916_2026_4715_MOESM8_ESM.pdf]

Additional file 8. Characteristics of included studies for survival changes over time

Characteristics of included studies lacking extractable molecular profiling data. Study periods are visually represented in Figure 3D (partitioned bar chart by enrollment era). Complete bibliographic references for all Study IDs are provided in the reference section at the end.

1st partition: before the Sciubba et al. 2007 study (the first to incorporate molecular profiling)

Study ID	Institute	Cohort span	Treatment	Age
Salzer 1973[30]	Universitätsklinik für Orthopädie und Unfallchirurgie, Wien	1965 – 1970	5/5 (100%) lateral vertebrectomy	59.2
Harrison 1985[31]	Bowman Gray School of Medicine of Wake Forest University, North Carolina	1977 – 1982	8/78 (10.3%) laminectomy<br>70/78 (89.7%) non-surgical treatments	54
Bach 1990[32]	University Hospitals Hvidovre and Glostrup, Copenhagen	1979 – 1985	NA	NA
Sørensen 1990[33]	Rigshospitalet, Copenhagen	1979 – 1985	NA	NA
Boogerd 1992[34]	Netherlands Cancer Institute (Antonivan Leeuwenhoek Ziekenhuis) Amsterdam	1984 – 1989	2/67 (3%) surgery, 2/67 (3%) no treatment, 63/97 (94%) chemotherapy, radiotherapy, hormone therapy	55.9
Maranzano 1992[35]	U.O. di Radioterapia Oncologica, Perugia	1986 – 1990	56/56 (100%) radiotherapy	57.8
Hill 1993[36]	Clinical Oncology Unit, Guy's Hospital, London	1976 – 1990	43/70 (61.4%) radiotherapy; 21/70 (30%) surgery; 6/70 (8.6%) supportive care	54
Tatsui 1996[37]	Osaka Medical College, Osaka	1980 – 1991	NA	52.6
Rachbauer 1996[38]	Universitätsklinik für Orthopädie, Innsbruck, Österreich	1972 - 1991	16/16 (100%) anterior approach, posterior approach, pedicular fixation, plate, acrylic cement implant	51.6
Shimizu 1992[39]	Kyoto University and Yoshikawa Hospital, Kyoto	1982 – 1991	4/4 (100%) decompression and stabilization	54.3
Buchelt 1996[40]	Universitätsklinik für Orthopädie, Wien	1970 – 1992	19/19 (100%) anterior decompression, majority	NA

Wagner 1996[41]	Paracelsus-Strahlenklinik, Osnabrück	1984 – 1995	12/12 (100%) radiotherapy	54.6
Okuyama 1999[42]	Fukuoka City Hospital, Kyushu University, Fukuoka	1990 – 1995	7/7 (100%) anterior or posterior approach, anterior fusion or instrumentation	53
Solberg 1999[43]	University of Tromsø, Norway	1986 – 1996	9 patients, radiotherapy with or without surgery	NA
Gokaslan 1998[44]	M. D. Anderson Cancer Center, Houston, Texas	1994 – 1997	10/10 (100%) transthoracic vertebrectomy	NA
Kasai 2007[45]	Mie University Graduate School of Medicine, Tsu, Mie	1991 – 1998	1/13 (7.7%) total spondylectomy; 12/13 (92.3%) palliative surgery	56.5
Sakaura 2004[46]	Osaka Minami National Hospital, Osaka	1993 – 1999	4/4 (100%) total en bloc spondylectomy	52.8
North 2005[47]	The Johns Hopkins University, Baltimore, Maryland	1990 – 2000	11/11 (100%) corpectomy or posterior instrumentation, laminectomy with fusion or placement of instrumentation	NA
Bilsky 2002[48]	Memorial Sloan-Kettering Cancer Center, New York	1994 – 2000	2/8 (25%) surgery; 6/8 (75%) radiotherapy	59.6

2nd partition: after the Sciubba et al. 2007 study (the first to incorporate molecular profiling)

Study ID	Institute	Cohort span	Treatment	Age
Sciubba 2007[49]	M. D. Anderson Cancer Center, Houston, Texas	1993 – 2001	87/87 (100%) posterior arthrodesis, posterior stabilization by implanting instrumentation	56.3
Schmidt 2006[50]	Odense Universitetshospital, Onkologisk Afdeling	1989 – 2003	54/54 (100%) surgery, radiotherapy, steroid	NA
Ulmar 2005[51]	Biometry and Medical Documentation, University of Ulm, Ulm	1984 – 2003	55/55 (100%) surgery	58.4
Ampil 2009[52]	Louisiana State University Health Sciences Center, Shreveport	1988 – 2005	105/105 (100%) radiotherapy	46.8

Gagnon 2007[53]	Georgetown University Medical Center, Washington DC	1995 – 2005	18/36 (50%) Cyberknife; 18/36 (50%) conventional external beam radiotherapy	55
Wibmer 2011[54]	Medical University of Graz, Medical University of Vienna	1998 – 2005	78/78 (100%) mixture of surgery and conservative	NA
Ampil 2010[55]	Louisiana State University Health Sciences Center, Shreveport	1990 – 2005	16/16 (100%) non-operatively treated, mainly by radiation	53.3
Pessina 2018[56]	Humanitas Cancer Center and Research Hospital, Rozzano	2004 – 2008	4/23 (17.4%) minimal resection; 11/23 (47.8%) curettage; 8/23 (34.8%) total tumorectomy	52.3
Switlyk 2015[57]	Norwegian Radium Hospital, Oslo University Hospital, Oslo	2007 – 2008	44/44 (100%) radiotherapy	NA
Tancioni 2011[58]	Istituto Clinico Humanitas, Milan	2004 – 2009	5/23 (21.8%) minimal resection; 18/23 (78.2%) curettage; 3/23 (13%) total tumorectomy	55
Walcott 2011[59]	Massachusetts General Hospital, Boston	2001 – 2009	10/15 (66%) anterior approach, corpectomy; 5/15 (33%) posterior approach, laminectomy	59.8
Weber 2014[60]	University of Lübeck, University Hospital Schleswig-Holstein	2009 – 2011	145/145 (100%) radiotherapy	62.9
Rades 2013[61]	University of Lübeck, University Hospital	1995 – 2011	510/510 (100%) radiotherapy	NA
Zadnik 2014[62]	The Johns Hopkins University, Baltimore, Maryland	2002 – 2011	43/43 (100%) single (anterior/posterior) approach or combined	56
Zakaria 2018[63]	Henry Ford Hospital, Detroit	2002 – 2012	118/118 (100%) stereotactic body radiation therapy	63.8
Sohn 2016[64]	Korean Health Insurance Review and Assessment Service database	2008 – 2012	978/978 (100%) mixture of no treatment, chemotherapy, surgery, and radiotherapy	NA

Oliveira 2015[65]	Hospital do Servidor Público Estadual de São Paulo	2010 – 2013	18/18 (100%) posterior approaches or combined anterior and posterior	NA
Azad 2016[66]	Stanford University School of Medicine, Stanford, California	2005 – 2013	5/5 (100%) metastasis to craniovertebral junction, stereotactic radiosurgery	60
Telera 2016[67]	National Cancer Institute, Rome	2005 – 2013	28/41 (68.3%) palliative surgery; 3/41 (7.3%) posterior stabilization; 10/41 (24.4%) vertebrectomy/somatectomy	58.5
Lee 2024[68]	Korean Health Insurance Review and Assessment Service	2011 – 2015	24/24 (100%) spinal instrumented fusion	NA
Huang 2018[69]	Fudan University Shanghai Cancer Center, Shanghai	2012 – 2015	2/2 (100%) total en bloc spondylectomy	45.5
Bernard 2017[70]	M. D. Anderson Cancer Center, Houston, Texas	2003 – 2013	25/25 (100%) spine stereotactic radiosurgery	56.2
Wright 2018[5]	Global Spinal Tumour Study Group	1991 – 2016	358/358 (100%) palliative decompressions, debulking, vertebrectomy	NA

3rd partition: studies published since 2020, but does not included studies with extractable molecular profiling data (see table 1)

Study ID	Institute	Cohort span	Treatment	Age
McCabe 2022[71]	Galway University Hospitals, Ireland	2010 – 2017	49/49 (100%) decompression, stabilization, kyphoplasty	NA
Rothrock 2021[72]	Memorial Sloan Kettering Cancer Center, New York	1998 – 2017	149/149 (100%) Separation surgery and radiotherapy, posterior instrumentation	NA
Terzi 2020[73]	IRCCS Istituto Ortopedico Rizzoli, Bologna	2009 – 2018	debulking, decompression, stabilization, vertebroplasty, minimal invasive spine surgery	58
Kato 2022[74]	Kanazawa Medical University, Kahoku	2006 – 2018	12/12 complete excision of spinal lesions	56.8
Chan 2022[75]	Northwestern University Feinberg School of Medicine,	2000 – 2019	49/49 (100%) single approach (posterior, anterior or lateral) or combined	56

	Chicago			
Rabah 2023[76]	Barrow Neurological Institute, St. Joseph's Hospital and Medical Center, Phoenix	2010 – 2020	15/131 (11%) surgery; 88/131 (64.7%) radiation	60.9
Knapp 2024[77]	Barnes-Jewish Hospital, St. Louis	2006 – 2020	15/15 (100%) anterior, posterior, or combined approach	NA
Ciérvide 2023[78]	Hospital Universitario HM Sanchinarro, HM Hospitales, Madrid	2010 – 2022	35/35 (100%) stereotactic body radiation therapy	NA
Taori 2024[79]	University of Pittsburgh Medical Center, Pennsylvania	2003 – 2023	99/99 (100%) spine stereotactic radiosurgery	59.3
